# Longitudinal change of selected human milk oligosaccharides and association to infants’ growth, an observatory, single center, longitudinal cohort study

**DOI:** 10.1371/journal.pone.0171814

**Published:** 2017-02-09

**Authors:** Norbert Sprenger, Le Ye Lee, Carlos Antonio De Castro, Philippe Steenhout, Sagar K. Thakkar

**Affiliations:** 1 Nestlé Research Center, Nestec Ltd., Vers-chez-les-Blanc, Switzerland; 2 Consultant Neonatologist, Department of Neonatology, Khoo Teck Puat-National University Children’s Medical Institute, National University Hospital, Singapore; 3 Assistant Professor, Department of Pediatrics, Yong Loo Lin School of Medicine, National University of Singapore, Singapore; 4 Nestlé Nutrition, Vevey, Switzerland; Indiana University Bloomington, UNITED STATES

## Abstract

**Background:**

Human milk is the recommended and sole nutrient source for newborns. One of the largest components of human milk is oligosaccharides (HMOs) with major constituents determined by the mother genotype for the fucosyltransferase 2 (FUT2, secretor) gene. HMO variation has been related with infant microbiota establishment, diarrhea incidence, morbidity and mortality, IgE associated eczema and body composition.

**Objectives:**

We investigated the (i) dependence of several major representative HMOs on the FUT2 status assessed through breast milk 2’Fucosyllactose (2’FL) and (ii) the relation of the 2’FL status with infant growth up to 4 months of life.

**Design:**

From an open observatory, single center, longitudinal cohort study with quantitative human milk collection at 30, 60, and 120 days postpartum from 50 mothers, who gave birth to 25 female and 25 male singleton infants, we collected a representative sample of human milk. We quantified the following 5 representative HMOs: 2’FL, Lacto-N-tetraose (LNT), Lacto-N-*neo*tetraose (LNnT), 3’Sialyllactose (3’SL) and 6’Sialyllactose (6’SL). We grouped the milk samples and corresponding infants according to the measured milk 2’FL concentrations at 30 days of lactation, which clustered around low concentrations (95% CI of mean 12–42 mg/L) and high concentrations (95% CI of mean 1880–2460 mg/L) with the former likely representing Secretor negative mothers. Infant anthropometric measures were recorded at birth, 1, 2 and 4 months of age. Relations among the quantified HMOs and the relation of the high and low 2’FL HMOs groups with infant growth parameters were investigated via linear mixed models.

**Results:**

The milk samples with low 2’FL concentration had higher LNT and lower LNnT concentrations compared to the samples with high 2’FL. The milk 3’- and 6’SL concentrations were independent of 2’FL. Over lactation time we observed a drop in the concentration of 2’FL, LNT, LNnT and 6’SL, especially from 1 to 2 months, while 3’SL remained at relatively constant concentration from 1 month onwards. Up to 4 months of age, we did not observe significant differences in body weight, body length, body mass index and head circumference of the infants who consumed breast milk with low or high FUT2 associated HMO concentrations and composition.

**Conclusions:**

Our findings on HMO concentrations over time of lactation and clusters based on 2’FL concentrations confirm previous observations and suggest that LNnT and LNT are ‘co-regulated’ with the FUT2 dependent 2’FL concentration, with LNnT showing a positive and LNT a negative relation. Further, our findings also suggest that the relatively substantial variation in HMOs between the high and low 2’FL clusters do not impact infant growth of either sex up to 4 months of age. The study was registered in www.ClinicalTrial.gov (NCT01805011).

## Introduction

Human milk is the sole nutrient source for newborn infants, adapted by evolution likely to provide both nutrition and protection [1]. Yet, in maternal milk, some nutrients such as vitamins and fatty acids vary depending on the dietary intake of the lactating mother, while others are primarily under genetic control [2]. To the best of the available knowledge, the major human milk oligosaccharides (HMOs) are determined primarily by the mother’s genotype.

HMOs represent one of the largest compound groups by mass after lactose and fat, with slightly higher concentrations than protein [3]. These generally non-digestible oligosaccharides are extensions of the milk sugar lactose and are brought about by the action of a series of glycosyltransferases such as those transferring N-acetyl-glucosamine, galactose, sialic acid or fucose. For the latter, fucosyltransferases FUT2 (secretor gene), FUT3 (Lewis gene) and probably FUT9 or others are implicated [4,5]. Of these, the former two are polymorphic with different alleles being responsible for the Secretor negative (*FUT2*-/-) and Lewis negative (*FUT3*-/-) glycosylation phenotypes not only in milk, but also on epithelial cell and mucosal surfaces.

The prevalence of functional FUT2 enzyme activity within the population varies strongly depending on geography [5]. In most geographic regions functional FUT2 alleles are predominant by over 70%, while in some regions of Asia and Africa non-functional FUT2 alleles were reported equally prevalent. Human microbial and viral interactions, in particular with pathogens but possibly also commensals or symbionts, are likely evolutionary forces that could have driven FUT2 dependent glycan loss and diversification in general [6]. Loss of functional FUT2 activity was associated with better resistance to some pathogens such as specific Norovirus genotypes and *Helicobacter pylori*, with the trade-off to have a higher risk for infections by other pathogens affecting respiratory, urinary tract or the gastro-intestinal systems. Furthermore, non-functional FUT2 genotypes were also associated with higher risk for type 1 diabetes, Crohn’s disease and neonatal sepsis (for review see [5]).

In women with a functional FUT2, 2’Fucosyllactose (2’FL) is the major milk oligosaccharide, followed by Lacto-N-fucopentaose I (LNFP I) and di-Fucosyllactose (diFL). These three HMOs depend on FUT2 and represent a good proxy to assess secretor status or the presence and absence of a functional FUT2 [7–10]. This manifests in measurable variation of specific human milk oligosaccharides and profiles [10,11]. Recently, the maternal milk FUT2 dependent oligosaccharide status was shown by association to affect the early establishment of a bifidobacteria dominated microbiota in breastfed infants [12,13] and possibly also the onset of IgE associated eczema in C-section born infants [14]. Further, other fucosyl-HMO might be related to allergic morbidity such as cow milk protein allergy [15]. The early bifidobacteria establishment and possibly also the breast milk FUT2 dependent oligosaccharides *per se* might lead to enhanced infant immune development and protection from infection [16–18]. Further, early life microbiota establishment was also shown to drive long-term morbidities such as asthma [19]. Recently, the concentration of a specific FUT2 dependent HMO, LNFP I, but not diFL or 2’FL, was reported to be associated with infant weight and body composition at 6 months of age [20].

Here, we set out to better understand the quantitative FUT2 dependent changes in representative HMOs over time of lactation and the impact of those changes for the early growth of the infants. To this end, we analyzed and quantified representative HMOs in relation to FUT2 dependent 2’FL from quantitative breast milk at 1, 2 and 4 months postpartum from a cohort of 50 mothers, who gave birth to 25 female and 25 male singletons. And, we explored if FUT2 dependent breast milk composition has an influence on growth, namely body weight, body length, head circumference and body mass index, in females and males up to 4 months of age.

## Materials and methods

### Study design, participants and milk sampling

The study was conducted in Singapore at the National University of Singapore. The participants provided written informed consent to participate in the study after receiving explanation and having read and understood the purpose of the study. The informed consent forms were approved by the National Healthcare Group (NHG) Domain Specific Research Boards (DSRB). This ethics board is made up of a group of independent reviewers and the processes had received AAHRP (Association for the Accreditation of Human Research Protection Programs) certification. Participants who did not understand English were provided with Chinese translated informed consent form as approved by the DSRB. The ethics approval reference for this study is NHG DSRB (domain B) 2011/00376. The protocol for this exploratory cohort study with quantitative collection of breast milk was reviewed and approved by NHG DSRB. The study was registered in www.ClinicalTrial.gov (NCT01805011).

We previously reported on lipid composition, which was the primary objective measurement of the breast milk samples from this same study cohort together with the secondary objectives of measuring lactose and energy content [21].

**Fig 1:**
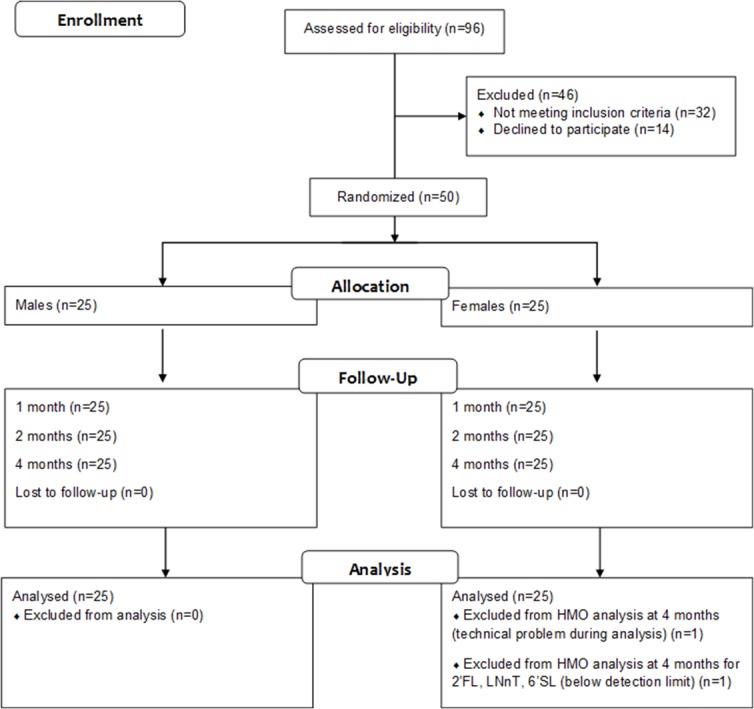
Study flow chart of the observational cohort study.

The study flow chart is depicted in Fig 1. Fifty mother-infant pairs were enrolled into the study between the infant’s birth and 1 month of age. Because of the exploratory nature of this trial, no formal sample size calculation was performed. Inclusion criteria were, birth at gestational age 37 to 42 weeks, mothers from 18 to 40 years old, mothers’ pre-pregnancy BMI (body mass index) between 20 kg/meter square (kg/m^2)^ and 29 kg/m^2^, mothers willing to breastfeed at least up to 4 months. Exclusion criteria were, pre-eclampsia, gestational diabetes, arterial hypertension above 140/90 mm Hg, and smoking. Visits were planned in the morning between 8:00 and 10:00 am to allow mothers to collect first morning expression either at home before the visit or during the visit at the hospital. Recruitment was from July 2011 to January 2012. Ninety six mother-infant pairs were screened for eligibility and 46 were excluded as 6 had gestational diabetes, 9 delivered premature at less than 37 weeks, 16 had a BMI below 20 kg/m^2^, 1 was over 40 years old, 5 declined to participate due to long distance from home to hospital, 9 were not suitable as they were unlikely to be motivated to continue breastfeeding until 4 months. For the visit at 4 months one mother was not able to provide breastmilk and for one breastmilk sample from the 4 months visit, we could not detect several of the HMOs that we intended to analyze due to unknown technical problem with the milk sample preparation. A total of 49 mothers provided 3 milk samples and a total of 48 milk samples was analyzed for all 5 representative HMOs at the 3 time points. As representative for the FUT2 dependent HMOs, we measured 2’FL, as representative of the sialylated HMOs, we measured 3’SL and 6’SL and as representative of the HMO core structures that can be further decorated with sialic acid or fucose, we measured LNT and LNnT.

A customized secure electronic Case Report Form database was used for data capture. Mothers were instructed to maintain a stable usual diet throughout the study.

For mothers’ demographic, dietary habit and health related data as well as data related to their infants’ birth (mode of delivery and parity) and breastfeeding habits (ever mixed feeding) were collected at enrollment.

For the infants, birth date, gender and gestational age were recorded at enrollment and anthropometry namely weight (kg), height (cm) and head circumference (cm) were recorded at birth and after enrollment to this study at the 1, 2 and 4 months visits. BMI was calculated.

The mothers provided breast milk samples (approximately 30 mL; at 30, 60, and 120 days postpartum). Samples were collected after full expression from one breast using a milk pump (Symphony Breastpump, Medela), while the baby was fed on the other breast to produce a satisfactory let-down. We made all efforts to collect complete feed that included fore-, mid-, and hind-milk as a representation of one feed and to avoid within feed variation of lipid and other nutrient contents. Approximately 30 mL aliquot was separated into two conical 15 mL polypropylene tubes for analysis and the rest was returned to the mother to feed the infant. Samples collected for research were stored at −80°C and shipped on dry ice for analyses to the Nestlé Research Center, Lausanne, Switzerland.

From a representative sample of quantitatively expressed breast milk we used the 2’FL concentrations measured in 30 day postpartum milk samples to group the mother infant pairs into those with low (considered Secretor negative) and high 2’FL concentrations.

### HMO analysis

For analysis by liquid chromatography, milk samples were defrosted, mixed and centrifuged for 20 min at 1700 × *g*. An aliquot of the skimmed milk was diluted 10 times with MilliQ water. All samples were analyzed thereafter by high performance anion exchange chromatography (HPAEC) coupled to a pulsed amperometry detector (ICS3000, Thermo Fischer Dionex, Sunnyvale, USA) in duplicate. Some samples were further diluted to allow for quantification. Briefly, samples were loaded onto a CarboPac PA1 analytical column (4x250mm; Thermo Fischer Dionex) without guard column at 25°C and a flow rate of 1 mL/min. Milk oligosaccharide separation and elution was achieved using the following gradient: For 18 min from 50mM NaOH to 135mM NaOH followed by a 7 min isocratic elution with 135mM NaOH and 7.5mM sodium acetate followed for the next 10 min with 135mM NaOH and a gradient of sodium acetate from 7.5mM to 12.5mM followed by a 10 min isocratic elution with 125mM NaOH and a gradient from 12.5 to 100mM sodium acetate followed by a 10 min wash step with 150mM NaOH and 250mM sodium acetate and a 10 min equilibration step at 50mM NaOH. For identification and quantification standard curves with authentic external standards were employed at concentrations of 1.25, 2.5, 5, 10, 30, 70 and 100 mg/l. Oligosaccharide standards were purchased from Dextra (UK). Eluents were prepared from 50% NaOH (J.T.Baker, UK) and sodium acetate (Fluka, CH). To control the HPAEC and to quantify the oligosaccharides the Chromeleon software package 6.8 was employed.

The detection of 2’FL, LNnT, LNT were shown to be linear in the concentration range of 1.25 to 100 mg/L. The detection of 6’SL and 3’SL were shown to be linear in the concentration range of 1.25 to 70 mg/L. Correlation coefficients were as follows: 2’FL (r^2^ = 0.9999); LNnT (r^2^ = 0.9997); LNT (r^2^ = 0.9998); 6’SL (r^2^ = 0.9979); 3’SL (r^2^ = 0.9988). Limit of detection were as follows: 2’FL: 0.13 mg/l; LNnT: 0.27 mg/l; LNT: 0.60 mg/l; 6’SL: 0.14 mg/l; 3’SL: 0.16 mg/l. Limit of quantification were as follows: 2’FL: 0.5 mg/l; LNnT: 0.9 mg/l; LNT: 2 mg/l; 6’SL: 0.5 mg/l; 3’SL: 0.5 mg/l. The method repeatability was measured by 6 repeated analysis of a human milk sample injected in triplicate. The following results were obtained: 2’FL (cv: ±1.2%), LNnT (cv: ±1.6%), LNT (cv: ±0.9%) 6’SL (cv: ±1.4%) 3’SL (cv: ±1.7%). Intermediate reproducibility was measured by analyzing a human milk sample in duplicate on 6 different days with an interval of time of several days between each analysis. The following results were obtained: 2’FL (cv: ±10%); LNnt (cv: ±7%); LNT (cv: ±4%); 6’SL (cv: ±11%); 3’SL (cv: ±10%). Trueness was determined by analyzing 6 times a human milk sample with low HMO content that was either supplemented with 150 mg/l or 300 mg/l 2’FL, LNnT, LNT, 3’SL and 6’SL and calculating the recovery of the added HMOs. The following results were obtained for the 150 and 300 mg/l additions, respectively: 2’FL: 102% and 96%; LNnT: 102% and 99%; LNT: 102% and 99%; 6’SL: 102% and 100%; 3’SL: 101% and 93%.

### Statistical analysis

Descriptive statistics and statistical analyses were done using an open source statistical software R version *3*.*0*.*1* (The R Foundation for Statistical Computing, Vienna, Austria).

A linear mixed model was used to assess the impact of 2’FL status (Low or High 2’FL) on the concentration of the other HMOs. A logarithmic transformation was performed on the HMO concentrations. 2’FL status, lactation stage, their interaction, mode of delivery and gender were declared as fixed effects and within subject variability was taken into account by declaring the subject as a random effect. No Imputation methods were used for missing values as there were very few such cases. Inclusion of parity, ethnicity, mothers’ BMI and age in the model were considered as they may have confounding effects, but these factors were discarded as no statistically significant effect was observed. Contrast estimates were then provided for each lactation stage using the *contrast* library to compare the differences between Low 2’FL and High 2’FL subgroups. The same model was used to assess the lactation stage effect comparing the consecutive stages for each of the HMOs.

A linear mixed model was also used to assess anthropometric differences between infants who were breastfed Low and High 2’FL breast milk. Lactation stage, 2’FL status, gender and their interaction were declared as fixed effects and within subject variability was taken into account by declaring the subject as a random effect. A similar model was done based on z-scores. Inclusion of breastfeeding habit (ever mixed feeding) in the model was considered as this may have confounding effect and was discarded as no statistically significant effect was observed.

## Results

### Baseline characteristics of the study cohort

The baseline characteristics of mothers and infants by low and high concentrations of 2’FL concentrations in breast milk are depicted in Table 1. Based on the 1 month post-partum milk samples we grouped the cohort into low 2’FL (mean 27 mg/L; 95% CI of mean 12–42 mg/L) and high 2’FL concentrations (mean 2170 mg/L; 95% CI of mean 1880–2460 mg/L). We had 16 mother infant pairs (32% of the study population) in the group with low 2’FL and 34 (68%) in the high 2’FL group. Both groups had an equal distribution between females and males and a similar distribution of gestational age, mode of delivery, parity and ethnicity. Only one mother was vegetarian. At birth, the anthropometry findings of both the male and female infants were similar from mothers with low 2’FL compared with those with high 2’FL. These anthropometry findings were within the means of the WHO child growth standards for each sex.

**Table 1:** Baseline data of mother-infant pairs in the Low- and High 2’Fucosyllactose groups.

		Low 2'Fucosyllactose		High 2'Fucosyllactose	
		n = 16	(%)	n = 34	(%)
Gender	Female	8	(50)	17	(50)
	Male	8	(50)	17	(50)
Gestational age (weeks)	37	2	(12)	1	(3)
	38	4	(25)	11	(32)
	39	5	(31)	9	(26)
	40	5	(31)	8	(24)
	41	0	(0)	5	(15)
Mode of delivery	Vaginal	12	(75)	19	(56)
	C-section	4	(25)	15	(44)
Weight at birth (kg) ^a^	Female	3.09 (*2*.*83–3*.*35*)		3.15 (*2*.*98–3*.*33*)	
	Male	3.46 (*3*.*20–3*.*71*)		3.38 (*3*.*21–3*.*55*)	
Body length at birth (cm) ^a^	Female	49.8 (*47*.*7–52*.*0*)		49.9 (*49*.*1–50*.*7*)	
	Male	51.0 (*49*.*6–52*.*4*)		51.0 (*49*.*9–52*.*0*)	
Head circumference at birth (cm) ^a^	Female	33.8 (*33*.*1–34*.*4*)		33.9 (*33*.*2–34*.*6*)	
	Male	34.5 (*33*.*8–35*.*2*)		33.6 (*33*.*1–34*.*0*)	
Parity	1	7	(44)	22	(65)
	2	5	(31)	10	(29)
	3	3	(19)	2	(6)
	4	1	(6)	0	(0)
Ethnicity	Caucasian	1	(6)	1	(3)
	Chinese	9	(56)	29	(85)
	Indian	3	(19)	3	(9)
	Malay	3	(19)	1	(3)
Mother food habits	Vegetarian	1	(6)	0	(0)
							
					

^a.^ mean (*95% CI of the mean*)

### Concentrations and interdependence of representative human milk oligosaccharides

**Fig 2:**
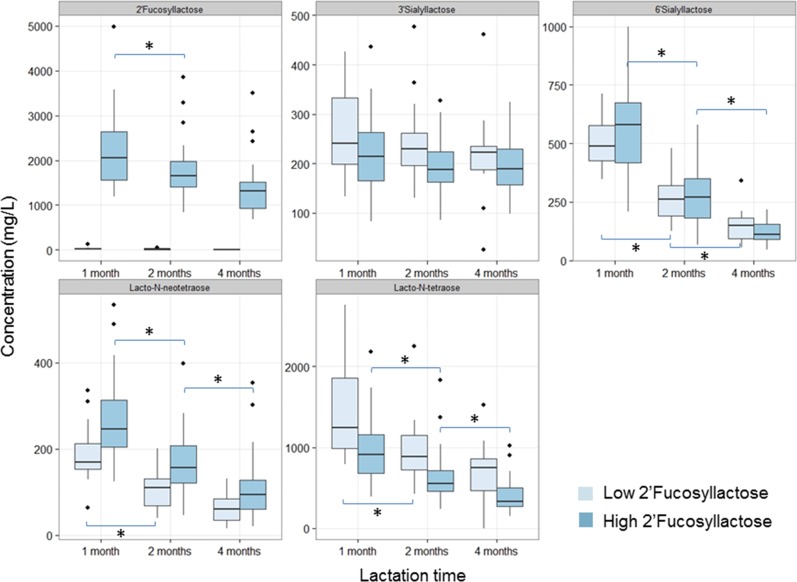
Box plot of HMO concentrations over first 4 months of lactation separated by group with Low- and High 2’Fucosyllactose in milk. (* indicates difference at a p<0.05; n = 50 for samples 1 and 2 months postpartum, n = 48 for samples 4 months postpartum).

We summarized the quantification of the analyzed HMOs over time of lactation by groups with low and high 2’FL concentrations in Table 2 and Fig 2.

**Table 2:** HMO concentrations (mg/L) in breast milk over time of lactation in milk of mothers from the Low- and High 2’Fucosyllactose groups.

		Low 2'Fucosyllactose						High 2'Fucosyllactose							
	*Months post partum*	n	Mean	*± SD*	Median	Min	Max	n	Mean	*± SD*	Median	Min	Max	estimate	*p value*
**2'FL**	1	16	26	*± 28*	15	7	122	34	2170	*± 832*	2053	1189	4990	105	0
** **	2	16	19	*± 11*	16	6	50	34	1764	*± 635*	1652	843	3863	102	0
** **	4	15	11	*± 5*	10	4	22	33	1376	*± 594*	1306	677	3505	126	0
**3'SL**	1	16	259	*± 88*	241	134	427	34	217	*± 74*	214	83	437	0.835	*0*.*109*
** **	2	16	243	*± 86*	229	130	477	34	195	*± 60*	188	87	328	0.803	*0*.*051*
** **	4	16	221	*± 90*	223	26	462	33	198	*± 59*	189	98	325	0.977	*0*.*838*
**6'SL**	1	16	496	*± 99*	487	347	714	34	561	*± 200*	581	208	1000	1.043	*0*.*724*
** **	2	16	265	*± 95*	263	125	478	34	280	*± 116*	269	68	579	0.996	*0*.*973*
** **	4	15	150	*± 71*	150	56	340	33	120	*± 45*	112	46	217	0.811	*0*.*082*
**LNnT**	1	16	189	*± 69*	170	64	336	34	263	*± 99*	247	124	534	1.492	*0*.*005*
** **	2	16	109	*± 47*	111	39	202	34	166	*± 72*	156	46	397	1.624	*0*.*001*
** **	4	15	66	*± 39*	60	16	131	33	108	*± 76*	94	20	353	1.739	*0*.*000*
**LNT**	1	16	1475	*± 607*	1248	790	2762	34	979	*± 394*	910	390	2185	0.666	*0*.*026*
** **	2	16	970	*± 432*	888	423	2254	34	633	*± 324*	551	236	1836	0.642	*0*.*015*
** **	4	16	703	*± 362*	753	3	1524	33	407	*± 200*	330	151	1024	0.757	*0*.*128*

The group with low 2’FL had median concentrations between 15 and 10 mg/L from 1 to 4 months of lactation, while the high 2’FL had a median of 2053 mg/L at 1 months, 1652 mg/L at 2 months and 1306 mg/L at 4 months (Table 2). As expected, in the group with high 2’FL concentrations, 2’FL was the most prominent HMO spanning a 95% CI of the mean at 1 months of lactation from 1880 to 2460 mg/L, at 2 months from 1543 to 1986 mg/L and at 4 months from 1165 to 1587 mg/L. With low 2’FL in milk, LNT became the most prominent HMO with a median concentration of 1248 mg/L at 1 month that dropped to 888 and 753 mg/L at 2 and 4 months respectively (Table 2). Over the 4 months lactation period milk LNT concentrations were significantly higher in the samples with low 2’FL as compared to those with high 2’FL. On the other hand, milk LNnT concentrations were lower in the samples with low 2’FL and higher in the high 2’FL milks. No such dependence on 2’FL was observed for the sialylated HMOs 3’SL and 6’SL (Table 2).

Over the 4 months lactation period, we found 3’SL at relatively constant concentrations both in the samples with low and high 2’FL (Fig 2). All other measured HMOs decreased over time in breast milk. In the group with low 2’FL, 6’SL reduced from a median of 487 mg/L at 1 month to 263 and 150 mg/L at 2 and 4 months respectively, while LNnT and LNT reduced only from 1 to 2 months postpartum. In the group with high 2’FL, 6’SL as well as LNnT and LNT reduced from 1 to 2 months and also from 2 to 4 months postpartum. The connections between these different HMOs are also illustrated in the correlation matrix depicted in S1 Fig. 2’FL inversely correlates with LNT concentrations and positively correlates with LNnT. The sialylated HMOs 3’SL and 6’SL correlate between them and 3’SL also correlates to LNT.

Together, the FUT2 status with low and high 2’FL as well as the observed relationships of 2’FL with LNT (negative correlation) and LNnT (positive correlation) show that breast milk varies substantially both in HMO composition and amounts of specific oligosaccharide structures over the 4 months lactation studied here.

### Effects of breast milk oligosaccharide composition on infant growth

**Fig 3:**
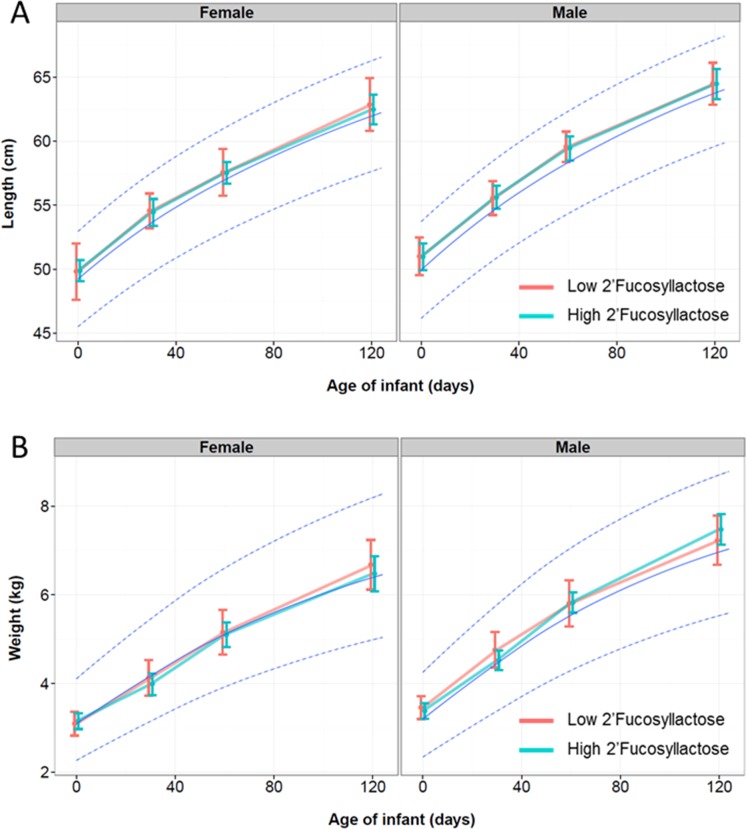
Body weight (in kg) and length (in cm) of infants over the first 4 months (120 days) from birth separated by gender and by those who were fed by mothers with Low 2’Fucosyllactose (Light red color; n = 16)) or High 2’Fucosyllactose (darker green color; n = 34) breast milk. The light blue line indicates the mean of the WHO child growth standard curve with the 95% CI. Means with standard deviation are depicted.

**Fig 4:**
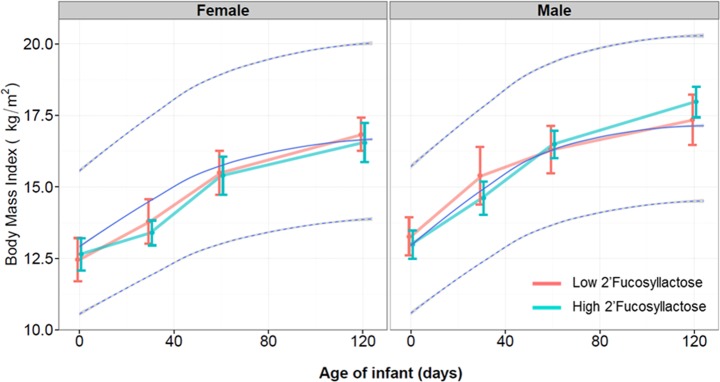
Body mass index (BMI, in kg/m^2^) of infants over the first 4 months (120 days) from birth separated by gender and by those who were fed by mothers with Low 2’Fucosyllactose (Light red color; n = 16)) or High 2’Fucosyllactose (darker green color; n = 34) breast milk. The light blue line indicates the mean of the WHO child growth standard curve with the 95% CI. Means with standard deviation are depicted.

**Fig 5:**
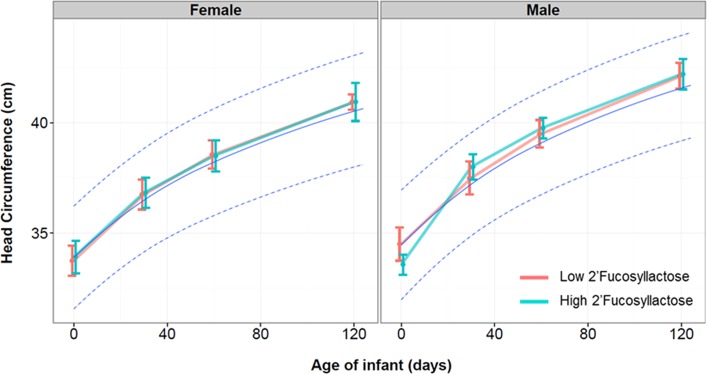
Head circumference (in cm) of infants over the first 4 months (120 days) from birth separated by gender and by those who were fed by mothers with Low 2’Fucosyllactose (Light red color; n = 16)) or High 2’Fucosyllactose (darker green color; n = 34) breast milk. The light blue line indicates the mean of the WHO child growth standard curve with the 95% CI. Means with standard deviation are depicted.

We analyzed the effect of FUT2 dependent HMO differences on infant growth of breastfed infants in terms of body weight and body length (Fig 3), body mass index (Fig 4) and head circumference (Fig 5). As illustrated, we plotted those growth parameters separated by females and males who were breastfed by mothers with low and high 2’FL type milk against the WHO growth curves for infants. Body length, weight, BMI and head circumference were not significantly affected by the breast milk types over time until 4 months.

Likewise, based on z-scores of body weight, body length, BMI and head circumference, we also did not observe any significant differences in the growth parameters of females and males who got either low or high 2’FL breast milk (data not shown).

Globally, we did not observe any significant impact of the low or high 2’FL breast milks on the measured infant growth parameters up to 4 months of age.

## Discussion

Here we show from quantitative, longitudinally collected breast milk samples, (i) a time dependent reduction of the major FUT2 dependent HMO 2’FL, the core structures LNT and LNnT and the sialylated HMO 6’SL, but not 3’SL and (ii) a relation of LNT and LNnT concentrations with 2’FL or FUT2 dependent HMOs. Since these FUT2 dependent breast milk composition changes are quite substantial, and because the affected HMOs were reported to affect early microbiota establishment [12,13], a key driver for infant health, and infant growth and body composition [20] we assessed in our cohort of breastfed infants whether their growth is affected by the breast milk FUT2 dependent HMO composition. Although preliminary due to the relatively low number of infants that we studied here, our findings suggest that early infant growth, at least up to 4 months, is independent of the qualitative and quantitative differences in the breast milk HMOs described here.

### FUT2-dependent HMO changes over time in breastmilk

The mother FUT2 (Secretor) status is probably the most important genetic factor affecting the breast milk glycosylation pattern and in particular the glycosylated lactoses commonly known as HMOs. In Secretor negative individuals the FUT2 encoded enzyme activity is strongly or completely reduced [22]. In lactating mothers the FUT2 polymorphism leads to missing or strongly reduced concentrations of 2’Fucosylated HMOs like 2’FL and LNFP I for example [7,23]. Interestingly, small amounts of 2’FL were seen in all our milk samples and small amounts of 2’fucosylation were also identified in milk of serologically tested Secretor negative women from Africa [23]. The majority of Secretor negative humans carry loss of function FUT2 mutations, while some mutations lead to missense and retain some enzyme activity [22,24,25]. Alternatively, the small amounts of 2’FL in milk of presumed Secretor negative mothers might also be due to some pathway redundancy. Interestingly, mothers in the high 2’FL group with the highest 2’FL concentrations and those with the lowest remained either highest or lowest throughout the 4 months of lactation. This indicates that the FUT2 expression levels are under tight control.

Previous studies had found that 6’SL in colostrum (1 to 3 days of lactation) was reported at about 370 mg/L and 3’SL at 300 mg/L [26], and in milk at 2–4 week of lactation 6’SL was about 380 mg/L and 3’SL 270 mg/L [27]. In our study, for milk at 1 month of lactation, we found 6’SL around 500 mg/L and 3’SL around 220 mg/L. These latter 3’SL values are similar to the reported concentration in colostrum and 2–4 week milk, indicating that 3’SL is expressed at relatively constant concentrations in very early and later milk. Further, in milk of mothers who gave preterm birth 3’SL concentrations were also relatively similar during the first month of lactation in the range of about 230 mg/L, while 6’SL was in the range of 500 to 700 mg/L [28]. As for 3’SL and 6’SL, our quantitative data on LNT and LNnT and 2’FL match very well those previously reported from different ethnic groups [7,26,29,30].

Depending on low or high 2’FL concentrations we saw significant differences in the concentrations of LNT and LNnT in milk confirming previous observations [31], but not seen for LNnT in one recent study [32]. While higher LNT concentrations were seen when 2’FL was low, we observed the contrary with LNnT, which was lower when 2’FL was low. This indicates that synthesis of LNT is independently regulated from 2’FL synthesis or FUT2 activity. On the other hand, formation of LNnT appears to be co-regulated with 2’FL synthesis or FUT2 activity. After lactose, LNT is the major acceptor substrate for FUT2 during HMO synthesis, leading to the formation of LNFP I [7]. After 2’FL, LNFP I is the second major 2’-fucosylated HMO [9,29]. Therefore, higher LNT in milk in the absence of 2’FL, or FUT2 activity, indicates that synthesis of the HMOs 2’FL and LNT is not co-regulated. On the other hand LNnT was lower in our study, when 2’FL or FUT2 activity was low. This might indicate co-regulated synthesis of 2’FL and LNnT or it could also be that LNnT is more readily used as acceptor substrate by other glycosyltransferases such as FUT3 [7], when FUT2 is low and GDP-fucose remains available as donor substrate. In line with this, Chaturvedi et al. [29] and Austin et al. [33] reported decreasing 2’FL and LNFP I over time of lactation concomitant with increasing 3-Fucosyllactose, a major 3’fucosylated HMO.

### Differences of breastmilk HMOs in relation to breastfed infants

As a consequence of the FUT2 polymorphism of mothers, infants naturally receive milk with different amounts and types of oligosaccharides. This leads to the evident question whether such a difference in breast milk composition is associated with specific infant morbidity, growth and developmental phenotypes. In a Mexican population higher concentrations of the FUT2 dependent oligosaccharides 2’FL and LNFP I in breast milk were associated with lower incidence of infectious diarrhea [18], indicating that the presence of FUT2 dependent HMOs might provide better protection from specific pathogens like *Campylobacter jejuni*, *E*. *coli* and norovirus [34]. Similarly, higher breast milk 2’-fucosylated HMOs, 2’FL and LNFP I, as well as non-2’-fucosylated HMOs were associated with lower infant mortality in HIV-exposed infants during lactation, but not thereafter [35]. In part, such observations might have been mediated via the establishing intestinal microbiota, which was also reported to be affected by the FUT2 dependent HMOs in breast milk [12,13]. FUT2 dependent variations of HMOs in breast milk, the sole and recommended nutrition for infants, might also associate with growth and development of the breastfed infant, for example through effects on the establishing gut microbiota. Alderete et al. [20] reported on an inverse relation between the concentrations of the FUT2 dependent LNFP I, but not 2’FL, in breast milk at 6 months and body weight, lean and fat mass of infants at 6 months. Further, LNnT also inversely related to body fat mass at 6 months of age.

In our study cohort we did not see any consistent effects of FUT2 positive or negative breast milk on infant growth up to 4 months of age. Notably, we had 33% of the infants who received breastmilk with low 2’FL throughout the studied 4 months indicating that these were Secretor negative milk and, although we did not quantify LNFP I in our study, milk with low or no 2’FL basically have no LNFP I, because both HMOs are synthesized by the same enzyme FUT2 (see also [9,20,29,36]). Therefore, 2’FL is a good proxy to group milks into low (Secretor negative) and high 2’Fucosyl-HMOs (secretor) that includes 2’FL, LNFP I and also diFL among other FUT2 dependent HMOs. Samples in our cohort with low 2’FL concentrations remained in the low concentration group at the 3 time points indicating that the low concentration is due to a genetic defect in FUT2 (e.g. Secretor negative genotype) and equally high 2’FL concentrations were also consistently high at 1, 2 and 4 months of lactation. Consequently, the categorical testing as done here for possible relations of low and high 2’FL milks with infant anthropometry is a stringent approach that is very unlikely to be biased by total breastmilk intake per day or variation of HMO concentration due to sampling and analytical variations. Based on infant growth curves and z-scores (body weight, body length, BMI and head circumference) we did not spot any indication that breast milk FUT2 dependent HMOs (including 2’FL, LNFP I, diFL) influence infant growth over the first 4 months of life. Yet, some variation in growth parameters was observed in our cohort. Although not statistically significant, but numerically visible, males from mothers with low 2’FL milk appeared to have a slightly higher BMI at 1 month, which was not seen any more at 4 months of age when they rather had a smaller BMI and body weight gain. Possible effects of FUT2 dependent HMOs on infant growth may not be apparent here, because our cohort represents an apparently healthy group of term-born infants. Further, we cannot exclude that changes in growth curves might only appear beyond 4 months of age.

## Conclusion

Together, although not powered to assess infant growth and only based on anthropometry and not body composition, we conclude from our exploratory observation study that FUT2 related alterations of breast milk HMOs composition as assessed through 2’FL concentrations, does not impact growth of breastfed infants during the first 4 months of life.

## Supporting information

S1 Fig Pearson correlation between HMO concentrations over first 4 months of lactation. 2FL, 2’Fucosyllactose; LNnT, Lacto-N-*neo*tetraose; LNT, Lacto-N-tetraose; 3SL, 3’Sialyllactose; 6SL, 6’Sialyllactose. **A**, at 1 month; **B**, at 2 months; **C**, at 4 months. Significant correlations (p<0.5) are in bold.
(TIF)

S2 Fig Breast milk concentration of 2’FL plotted against LNT (upper panels) and LNnT (lower panels) at 1, 2, and 4 months of lactation with linear regression line. (TIF)

S1 File Clinical trial protocol. (PDF)

S2 File Consort checklist. (DOC)

S1 Table Raw data table. (PDF)

## Abbreviations

FUT2: Fucosyltransferase 2
2’FL: 2’Fucosyllactose
LNnT: Lacto-N-neotetraose
LNT: Lacto-N-tetraose
3’SL: 3’Sialyllactose
6’SL: 6’Siayllactose
HMOs: human milk oligosaccharides
