# Supplementary figures and images for: Longitudinal change of selected human milk oligosaccharides and association to infants’ growth, an observatory, single center, longitudinal cohort study
Source: PLoS One. 2017 Feb 9;12(2):e0171814. doi: 10.1371/journal.pone.0171814 (PMC5300226; doi:10.1371/journal.pone.0171814)

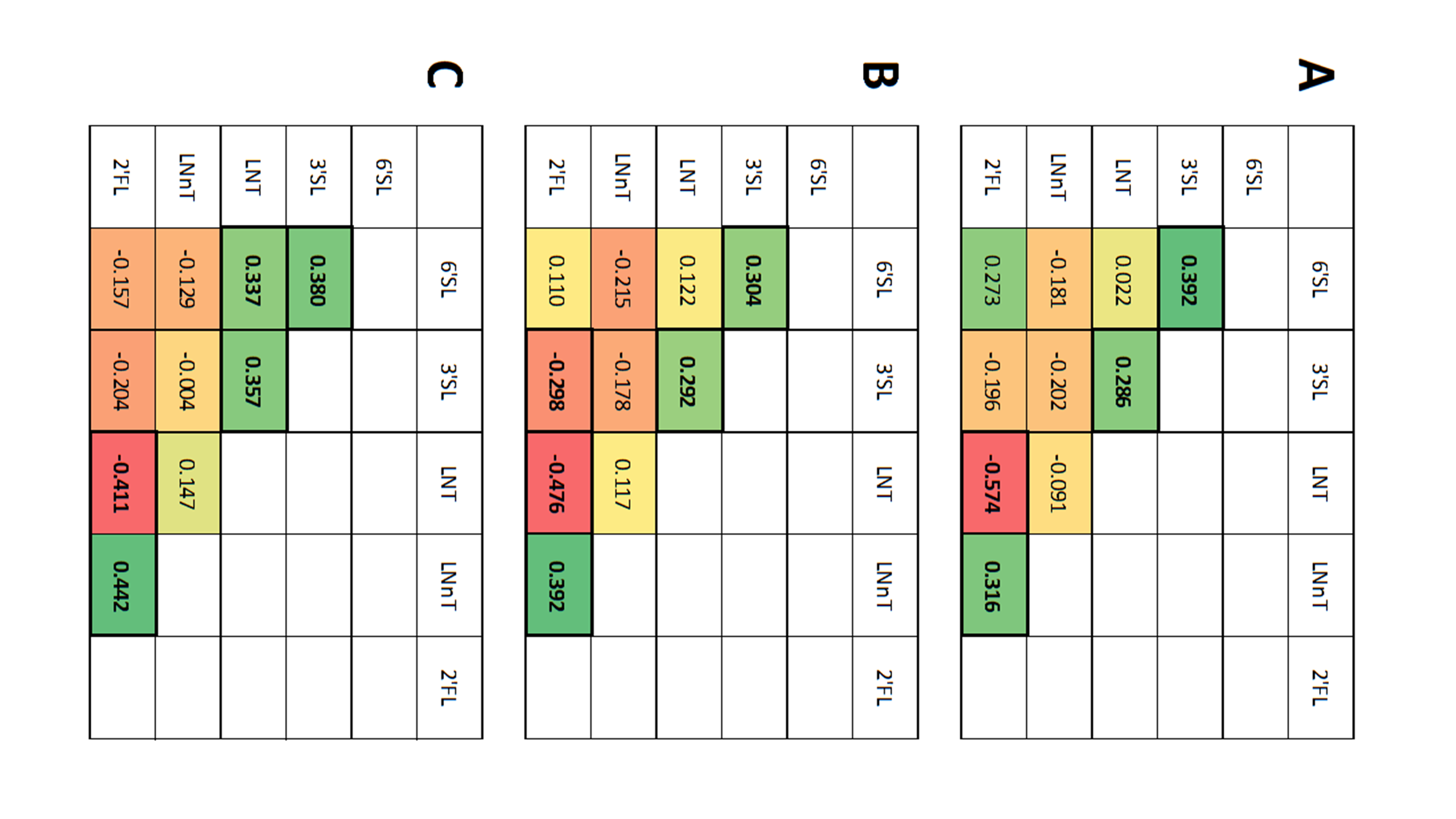

Supplement: S1 Fig — 2FL, 2’Fucosyllactose; LNnT, Lacto-N-neotetraose; LNT, Lacto-N-tetraose; 3SL, 3’Sialyllactose; 6SL, 6’Sialyllactose. A, at 1 month; B, at 2 months; C, at 4 months. Significant correlations (p<0.5) are in bold. (TIF) [file pone.0171814.s001.tif]

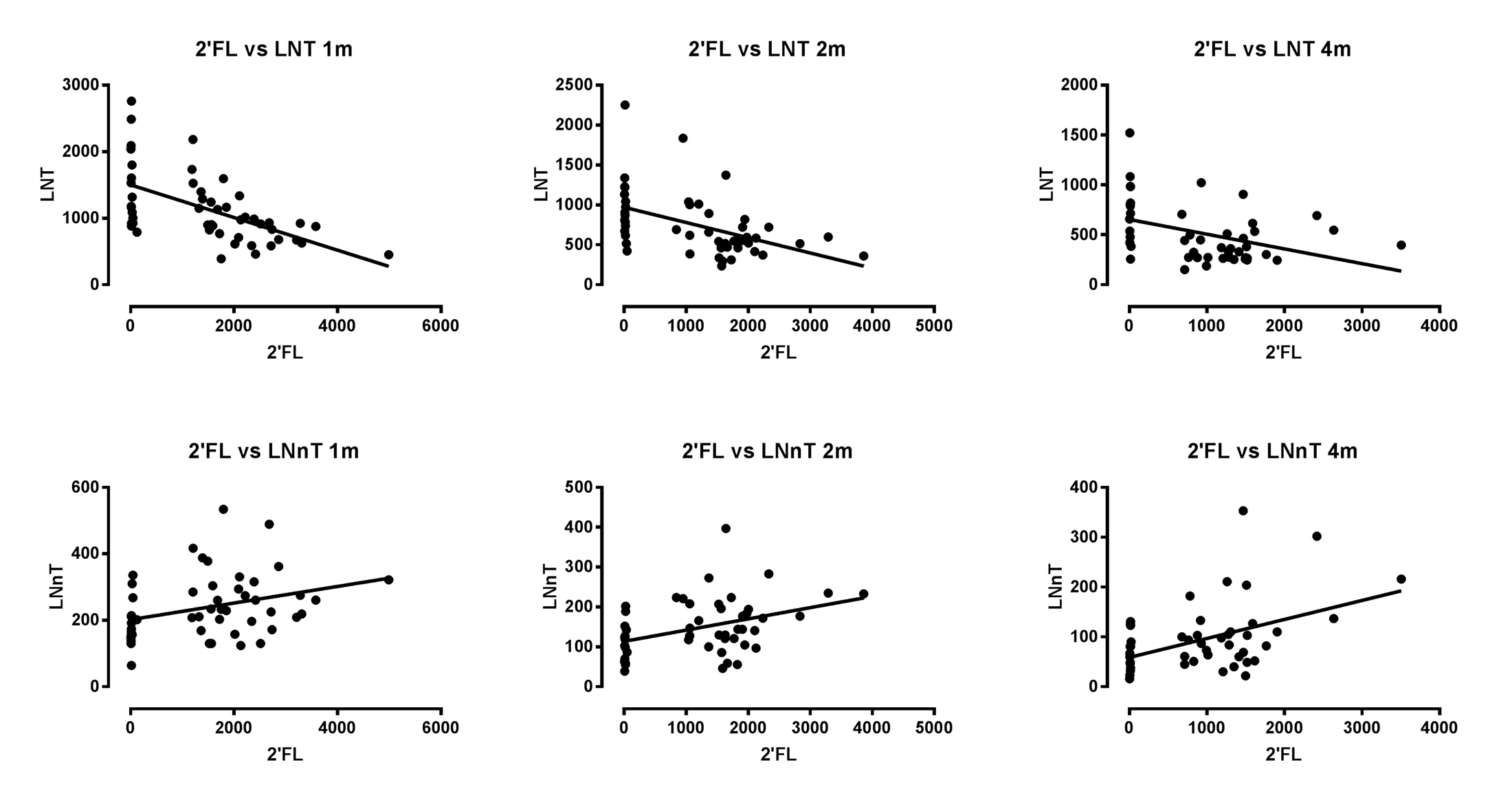

Supplement: S2 Fig — (TIF) [file pone.0171814.s002.tif]
