# Supplementary material for: Longitudinal change of selected human milk oligosaccharides and association to infants’ growth, an observatory, single center, longitudinal cohort study
Source: PLoS One. 2017 Feb 9;12(2):e0171814. doi: 10.1371/journal.pone.0171814 (PMC5300226; doi:10.1371/journal.pone.0171814)
Supplement: S1 File — (PDF) [file pone.0171814.s003.pdf]

STUDY PROTOCOL

Study N°	11.08.INF
NPDI Code N°	DNUT-100082

Study on normal ranges of phospholipids & gangliosides in breast milk of healthy mothers

Project Excellence

Date: **September 21, 2011**
Version: **AMENDED**
N° **1**

Sponsor Nestec Ltd
Avenue Nestlé 55
CH-1800 Vevey
Switzerland

CONFIDENTIAL

Nestlé Research Center is certified ISO 9001:2008 for Research on nutrition and food, including preparation of test products and the organization and conducting of clinical trials

TABLE OF CONTENTS

1 COORDINATING INVESTIGATOR SIGNATURE PAGE 5
2 SPONSOR TEAM SIGNATURES PAGE 7
3 STUDY SITES 8
4 STUDY CONTACT INFORMATION 8
5 ABBREVIATIONS - DEFINITIONS 9
6 SYNOPSIS 10
7 STUDY PLAN 12
8 INTRODUCTION 13
9 OBJECTIVES OF THE TRIAL 14
9.1 Primary objective 14
9.2 Primary outcome 14
9.3 Secondary Objectives 14
9.4 Secondary outcomes 14
10 TRIAL DESIGN 15
10.1 Type of trial 15
10.2 Subjects, groups and centers 15
10.3 Expected study duration 15
11 STUDY POPULATION 15
11.1 Description 15
11.2 Subject inclusion criteria 15
11.3 Subject exclusion criteria 15
11.4 Subject withdrawal criteria 15
12 STUDY PRODUCT 15
12.1 Study product description 15
12.2 Study product administration 16
12.3 Study product handling 16
12.3.1 Storage and distribution 16
12.3.2 Study product accountability and reconciliation 16
12.4 Concomitant diet and treatment 16
12.4.1 Permitted concomitant diets / treatments / medications 16
12.4.2 Unauthorized concomitant diets / treatments / medications 16
12.4.3 Concomitant diets / treatments / medications record 16
13 ASSESSMENT OF EFFICACY 16
14 ASSESSMENT OF SAFETY 16

CONFIDENTIAL
The contents of this document constitute a trade secret and the intellectual property rights thereto belong to Nestlé S. A., Vevey, Switzerland.
This document may not be reproduced or disclosed to third parties without prior authorisation.

15 CONDUCT OF THE TRIAL....16
15.1 Subject recruitment....16
15.2 Baseline....16
15.3 Visits....17
15.3.1 Visit 1 (1 month +/- 5 days)....17
15.3.2 Visit 2 (2 months +/- 5 days)....17
15.4 Final Visit (4 months +/- 7 days)....17
15.5 Biological samples....18
15.6 Follow-up....20

16 DATA MANAGEMENT....20
16.1 Data collection....20
16.2 Computerized edit checks....20
16.3 Access rights....21
16.4 Audit trail....21

17 STATISTICS....21
17.1 Background....21
17.2 Effects to be estimated....21
17.3 Interim Analysis....21
17.4 Sample size calculations....21
17.5 Randomization....21
17.6 Datasets to be analyzed....21
17.6.1 Full analysis dataset....21
17.6.2 PP analysis dataset....21
17.7 Statistical analysis....21
17.7.1 Primary analysis....21
17.7.2 Key secondary analyses....22

18 HANDLING OF ADVERSE EVENTS....22
18.1 Definition: Adverse event....22
18.2 Intensity....22
18.3 Seriousness....22
18.4 Unexpected or expected SAE....23
18.5 Relation with test product....23
18.6 Reporting and Documentation....23
18.7 Follow up....24
18.8 Notification....24
18.9 Reporting....24

19 LEGAL AND ETHICAL PREREQUISITES....24
19.1 Legal requirements....24
19.2 Ethical aspects....24
19.2.1 Protection of the subject's confidentiality....24
19.2.2 Informed consent....24

CONFIDENTIAL
The contents of this document constitute a trade secret and the intellectual property rights thereto belong to Nestlé S. A., Vevey, Switzerland.
This document may not be reproduced or disclosed to third parties without prior authorisation.

19.2.3 Ethics committee approval 25
19.2.4 Declaration of Helsinki 25

20 QUALITY CONTROL AND QUALITY ASSURANCE 25
20.1 Monitoring 25
20.2 Source documents 25
20.3 Quality Control 25
20.3.1 Quality control of essential documents 25
20.3.2 Co-monitoring 25
20.4 Audits and inspections 26
20.5 Responsibilities of investigator 26

21 STUDY END PROCEDURES 26
21.1 Premature termination of study 26
21.2 Termination of study 26

22 REFERENCES 28

23 APPENDICES 30

CONFIDENTIAL
The contents of this document constitute a trade secret and the intellectual property rights thereto belong to Nestlé S. A., Vevey, Switzerland.
This document may not be reproduced or disclosed to third parties without prior authorisation.

1 COORDINATING INVESTIGATOR SIGNATURE PAGE

COORDINATING INVESTIGATOR	
Dr. LEE Le Ye<br>MBBS, Mmed (Paediatrics) Singapore, MRCPCH<br>Consultant, Department of Neonatology,<br>Assistant Professor, Yong Loo Lin School of Medicine,<br>National University Healthcare System<br>5 Lower Kent Ridge Road<br>Singapore 119074<br>Tel : +65 67724574<br>Fax : +65 68725971<br>Le_Ye__Lee@nuh.com.sg	Date:<br><br>Signature

By my signature, I agree to supervise and oversee the conduct of this study and to ensure its conduct is in compliance with the protocol, informed consent, IRB/EIC procedures, instructions from Nestlé representatives, the Declaration of Helsinki, ICH Good Clinical Practices guidelines and the applicable local regulations governing the conduct of clinical studies.

Department of Pediatrics, Yong Loo Lin School of Medicine, National Univeristy of Singapore

CONFIDENTIAL
The contents of this document constitute a trade secret and the intellectual property rights thereto belong to Nestlé S. A., Vevey, Switzerland.
This document may not be reproduced or disclosed to third parties without prior authorisation.

Investigator(s) signature(s) page

PRINCIPAL INVESTIGATOR

Dr. LEE Le Ye
MBBS, Mmed (Paediatrics) Singapore, MRCPCH
Consultant, Department of Neonatology,
Assistant Professor, Yong Loo Lin School of Medicine,
National University Healthcare System
5 Lower Kent Ridge Road
Singapore 119074
Tel : +65 67724574
Fax : +65 68725971
Le_Ye _Lee@nuh.com.sg

Date:

Signature

SUB- INVESTIGATOR

Dr. Yvonne Ng Peng Mei, Consultant,
Department of Neonatology, National University Hospital,
Associate Professor,
Yong Loo Lin School of Medicine,
National University Hospital.

Date:

Signature

I have read this protocol and agree that it contains all necessary details for carrying out this study. I will conduct the study as outlined herein and will complete the study within the time designated.

I will provide copies of the protocol and all pertinent information to all individuals responsible to me who assist in the conduct of this study. I will discuss this material with them to ensure they are fully informed regarding the study product and the conduct of the study.

I will use only the informed consent form approved by the sponsor or its representative and will fulfill all responsibilities for submitting pertinent information to the Independent Ethics Committee (IEC) responsible for this study.

I agree that the sponsor or its representatives shall have access to any source documents from which case report form information may have been generated.

CONFIDENTIAL
The contents of this document constitute a trade secret and the intellectual property rights thereto belong to Nestlé S. A., Vevey, Switzerland.
This document may not be reproduced or disclosed to third parties without prior authorisation.

2 SPONSOR TEAM SIGNATURES PAGE

PROJECT MANAGER

Liên-Anh TRAN
Nestlé Nutrition
Av. Reller 22
1800 Vevey
Switzerland
Tel: +41 21 924 2331
lien-anh.tran@nestle.com

Date:

Signature

BIOSTATISTICIAN

Andreas Rytz
Nestlé Research Centre
Bioanalytical Science
PO Box 44, CH-1000 Lausanne 26
Switzerland
Tel: +4121 785 8762
andreas.rytz@rdls.nestle.com

Date:

Signature

DATA MANAGER

Emilie BA
Nestlé Research Centre
Clinical Development Unit
PO Box 44, CH-1000 Lausanne 26
Switzerland
Tel: +41 21 785 8068
Fax: +41 21 785 8556
emilie.ba@rdls.nestle.com

Date:

Signature

CLINICAL PROJECT MANAGER

Julie CHAMBARD
Nestlé R&D Center (PTE) LTD
N°29 Quality Road
Singapore 618802
Tel: +65 68900 100
julie.chambard@rdsg.nestle.com

Date:

Signature

SPONSOR

Nestec SA represented by
Philippe STEENHOUT, MD
Assistant Vice President
Av. Reller 22
1800 Vevey
Switzerland
Tel: +41 21 924 29 74
philippe.steenhout@nestle.com

Date:

Signature

CONFIDENTIAL
The contents of this document constitute a trade secret and the intellectual property rights thereto belong to Nestlé S. A., Vevey, Switzerland.
This document may not be reproduced or disclosed to third parties without prior authorisation.

3 STUDY SITES

COUNTRY / CITY	INVESTIGATOR NAME	INSTITUTION, PHONE, E-MAIL
SINGAPORE	Dr. Le Ye LEE	Dr. LEE Le Ye<br>Department of Neonatology,<br>Assistant Professor, Yong Loo Lin School of Medicine,<br>National University Healthcare System<br>5 Lower Kent Ridge Road<br>Singapore 119074<br>Tel : +65 67724574<br>Fax : +65 68725971<br>Le_Ye__Lee@nuh.com.sg

4 STUDY CONTACT INFORMATION

ROLE	NAME	ADDRESS, PHONE, E-MAIL
Central Laboratory	NA	
Monitors	Choo Hoffmann	centris@westnet.com.au
Clinical Research Organization	NA	
Central storage	NA	
Medical Safety Officer	Philippe STEENHOUT	Nestlé, Av. Reller 22<br>CH-1800 Vevey<br>+41 21 924 29 74<br>philippe.steenhout@nestle.com
Clinical Safety Manager	Julien DEY	Nestlé Research Centre, CDU<br>PO box 44<br>CH-1000 Lausanne 26<br>+41 21 785 95 10<br>julien.dey@rdls.nestle.com
Product Manager	NA	

CONFIDENTIAL
The contents of this document constitute a trade secret and the intellectual property rights thereto belong to Nestlé S. A., Vevey, Switzerland.
This document may not be reproduced or disclosed to third parties without prior authorisation.

5 ABBREVIATIONS - DEFINITIONS

**AE**	Adverse Event
**CRA**	Clinical Research Associate (synonym: monitor)
**CRF**	Case Report Form
**CPM**	Clinical Project Manager
**GCP**	Good Clinical Practice
**IEC**	Independent Ethic Committee
**IRB**	Institutional Review Board
**ITT**	Intent-To-Treat
**MP**	Method and Procedure:<br>**(**providing all the information necessary to carry out **activities** efficiently, safely and in an authorized manner).
**NRC**	Nestlé Research Centre
**PP**	Per-Protocol
**SAE**	Serious Adverse Event
**SOP**	Standard Operating Procedure:<br>(providing all the information necessary to use **equipment** efficiently, safely and in an authorized manner)
**SUSAR**	Serious Unexpected Suspected Adverse Reaction<br>(see definition in chapter 19.4.)
**TMF**	Trial Master File

CONFIDENTIAL
The contents of this document constitute a trade secret and the intellectual property rights thereto belong to Nestlé S. A., Vevey, Switzerland.
This document may not be reproduced or disclosed to third parties without prior authorisation.

6 SYNOPSIS

Primary objective and outcome:

This trial is undertaken to better understand the ranges of functional components normally found in human milk. This will strengthen the scientific knowledge on breast-milk composition as a “gold standard” and further support the development of infant formulae providing phospholipids & gangliosides in ranges matching closely the mother’s milk physiological levels, and thereby delivering the same functionalities.

The primary objective of this trial is to determine the levels of phospholipids and gangliosides normally found in the breast-milk of healthy lactating mothers, using state-of-the-Art validated methods (already developed at the Nestlé Research Centre).

- ✓ Phospholipids and gangliosides will be analysed in a 10 mL sample of fully expressed mature milk, corresponding to a complete feed, and taken at 1st, 2nd and 4th months.

Secondary objectives and outcomes:

Secondary objectives include the measure of the profile and quantity of oligosaccharides found in human milk, as well as the generation of further knowledge & data on the potential relations between human milk composition and e.g. dietary habits, ethnicity, etc.

- ✓ HMO analyses will be conducted on a 10 mL sample of fully expressed mature milk, corresponding to a complete feed, and taken at 1st, 2nd and 4th months.

Design:

This is an open, single-centre, 1 group study.

Number of subjects:

Nb of mothers needed = 50 (25 male babies/ 25 female babies)

Description of subjects and key criteria for inclusion/exclusion:

Inclusion criteria

- Gestational age between 37 and not above 42 weeks
- Baby to be enrolled between birth and V1
- 18 years old ≤ Mother ≤ 40 years old
- 18.5 ≤ pre-pregnancy BMI ≤ 29
- Mothers willing to breastfeed for the first 4 months

Exclusion criteria

- Gestational diabetes
- HTA < 140/90
- Mothers who are smokers while breast-feeding

Additional information

- Mothers of twins can be included
- Specify the breast from which milk has been extracted (2 samples of 10 mL are needed).
- Visits should be planned between 08:00-10:00 am (letting the flexibility to the mother of collecting the first morning expression either at home or in the hospital. Most breast-fed babies will be up at 7am and had done their feed at home).
- Recruitment start = July 2011.

CONFIDENTIAL
The contents of this document constitute a trade secret and the intellectual property rights thereto belong to Nestlé S. A., Vevey, Switzerland.
This document may not be reproduced or disclosed to third parties without prior authorisation.

Product(s) to be tested:
Not Applicable

Amount, dosage, route of administration, duration of study product:
Not Applicable

Study procedure:
Subjects selected during the screening visit will follow a 4-month study. Primary and secondary outcomes will be measured at 1, 2 and 4 month(s).

Mothers study plan:		**V_1**	**V_2**	**V_3**
Month(s)		1	2	4
Mother's information	Weight<br>- at birth<br>- before pregnancy	X		
	Height			
	Mode of delivery			
	Parity			
	Ethnic Origin			
	Blood group			
	Food habits (vegetarian or not)			
Baby's anthropometry <u>*at birth*</u> (Weight, Length and Head circumference)		X		
Baby's anthropometry		X	X	X
3-day Food diary for mother		X	X	X
Breast milk sample		X	X	X

Data management and statistical analysis:
This study uses a customized secure electronic Case Report Form (eCRF) database.

The primary analysis consists in establishing normal ranges for phospholipids and gangliosides in human milk at 1, 2 and 4 months after delivery.

Key secondary analyses consist in establishing similar normal ranges for oligosaccharides, estimating significance of trends over time (1 to 4 months) and exploring relations with potential confounders (e.g. mother's age, blood group, ethnicity, BMI and food habits, as well as infant's gender or BMI).

CONFIDENTIAL
The contents of this document constitute a trade secret and the intellectual property rights thereto belong to Nestlé S. A., Vevey, Switzerland.
This document may not be reproduced or disclosed to third parties without prior authorisation.

7 STUDY PLAN

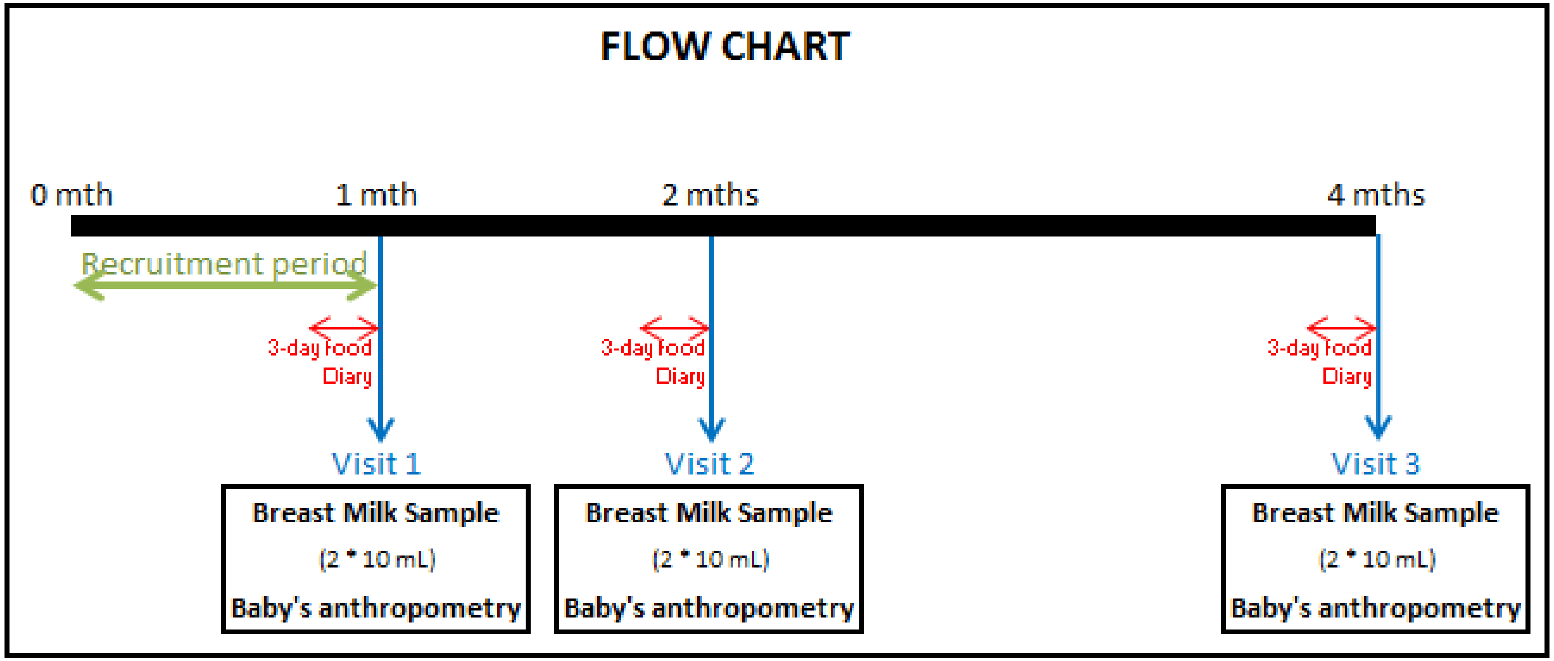

CONFIDENTIAL
The contents of this document constitute a trade secret and the intellectual property rights thereto belong to Nestlé S. A., Vevey, Switzerland.
This document may not be reproduced or disclosed to third parties without prior authorisation.

8 INTRODUCTION

Breast milk is the most nutritionally sound food for babies. It consists of nutrients, such as proteins, lipids, carbohydrates, minerals, vitamins, and trace elements that the babies need to grow healthy. It also contains immune-related components such as sIgA, leukocytes, oligosaccharides, lysozyme, lactoferrin, interferon-g, nucleotides, cytokines, and others. Several of these compounds offer passive protection in the gastrointestinal tract and to some extent in the upper respiratory tract, preventing adherence of pathogens to the mucosa and thereby protecting the breast-fed infant against invasive infections. Human milk also contains essential fatty acids, enzymes, hormones, growth factors, polyamines, and other biologically active compounds, which may play an important role in the health benefits associated with breast-feeding (ESPGHAN, 2009).
In cases when a woman is unable to feed her child with her own milk or cannot supply enough milk, or when breastfeeding is contraindicated, babies may be fed infant formula.
In order to support the development of infant formulas matching closely the composition and functions of human breast milk, this trial is undertaken to better understand the ranges of functional components normally found in human milk. In particular, this project aims to develop an infant formula providing phospholipids & gangliosides in ranges matching closely the mother's milk physiological levels, and thereby delivering the same functionalities.

Phospholipids
The ESPGHAN committee considered that phospholipids have key functions in cellular signal transduction and in solubilizing lipophilic compounds in the human intestinal lumen, and may be added to infant formula based on the levels observed in human breast milk (Koletzko et al., 2005). Phospholipids (e.g. phosphatidylcholine, phosphatidylserine, sphingomyelin) are reported to have effects on neuronal development, protection of the human gastrointestinal mucosa and recovery from toxic attack, synthesis and transmission of neurotransmitters important to memory, and might also be involved in brain development. Furthermore, some phospholipids are digested in the gastrointestinal tract to compounds that might possess antimicrobial activity. There is also evidence that milk phospholipids exert a strong gastroprotective role in humans, particularly in the duodenal mucosa (Dewettinck et al., 2008).

Gangliosides
The role of gangliosides in human milk continues to be a subject of research and discussion in the fields of paediatric nutrition and glycobiology. The distribution profile of milk gangliosides selectively changes during lactation, suggesting that gangliosides may participate in physiological processes that take place in the early development of infants. Dietary gangliosides may have an important role in the modification of intestinal microbiota and the promotion of intestinal immunity development in the neonate, and consequently in the prevention of infections during early infancy (Rueda, 2007).

Oligosaccharides
Human milk oligosaccharides (HMO) are the third largest milk compound group based on mass present in human milk. Over 130 individual structures have been identified to date but the functional implications of this diversity are not yet known (Kunz et al., 2000). The majority of the oligosaccharides in milk are not digestible by human infants. This apparent paradox raises obvious questions about the functions of these oligosaccharides and how their diverse molecular structures affect their functions. The nutritional function that is most frequently attributed to milk oligosaccharides is to serve as prebiotics – a form of indigestible carbohydrate that is selectively fermented by desirable gut microbiota (German et al., 2008).

The primary objective of this trial is to determine the levels of phospholipids and gangliosides normally found in the breast-milk of healthy lactating mothers, using state-of-the-Art validated methods (already developed at the Nestlé Research Centre) and following a thorough sampling protocol (fully expressed mature milk corresponding to a complete feed).

CONFIDENTIAL
The contents of this document constitute a trade secret and the intellectual property rights thereto belong to Nestlé S. A., Vevey, Switzerland.
This document may not be reproduced or disclosed to third parties without prior authorisation.

Secondary objectives include the measure of the profile and quantity of oligosaccharides found in human milk, as well as the generation of further knowledge & data on the potential relations between human milk composition and e.g. dietary habits, ethnicity, etc.

This trial will be conducted in compliance with the protocol, GCP and the applicable regulatory requirement(s).

9 OBJECTIVES OF THE TRIAL

9.1 Primary objective

A literature search allowed us to identify a few papers dealing with phospholipids, gangliosides, and cholesterol contents in mature human milk. However, in several papers, either the human milk collection method was not specified, or only foremilk was collected, whereas the most reliable information would come from a complete breast milk expression. In addition, analytical methods used to quantify nutrients in human milk were poorly described, providing little information on their performance (Bitman et al., 1984 & 1986; Collins et al.,1989; Huston et al., 1986; Martin-Sosa et al., 2004; Pan et al., 1999; Rueda et al., 1996; Huisman et al., 1996; Lammi-Keefe et al., 1990; Peterson et al., 1998).

The primary objective of this trial is to determine the levels of phospholipids and gangliosides normally found in the breast-milk of healthy lactating mothers, using state-of-the-Art validated methods (already developed at the Nestlé Research Centre) and following a thorough sampling protocol (fully expressed mature milk corresponding to a complete feed).

9.2 Primary outcome

The contents of breast milk adapt to the infant's needs throughout his development (colostrum has a different composition than transitional or milk) and also changes over the course of a breastfeeding session (watery milk at the beginning of the feed, then fat and energy contents increase to reach highest levels at the end of the feed). Therefore:

- ✓ Phospholipids and gangliosides will be analysed in a 10 mL sample of fully expressed mature milk, corresponding to a complete feed, and taken at 1st, 2nd and 4th months.

9.3 Secondary Objectives

Human milk oligosaccharides (HMO) are the third largest milk compound group based on mass present in human milk. Reported amounts in mature milk vary between 5 and 10 g/L, and over 130 individual structures have been identified to date (Kunz et al., 2000). Structural diversity and amount of specific HMO depend on the Lewis blood group and secretor status (Erney et al., 2000; Chaturvedi et al., 2001; Sjögren et al., 2007). However, very little is known about HMO content and structural variations within a breast feed and linked to ethnicity, nutritional habits, and time of lactation.

Secondary objectives include the measure of the profile and quantity of oligosaccharides found in human milk, as well as the generation of further knowledge & data on the potential relations between human milk composition and e.g. dietary habits, ethnicity, etc.

9.4 Secondary outcomes

For many HMO a very large range is reported. This variation might be in part due to inappropriate sampling procedures during a feed, nutritional habits and Lewis and secretor status of the mother (Leo et al., 2009; Asakuma et al., 2007 & 2008). Such variables are generally insufficiently described in the literature on HMO in breast milk. Furthermore, analytical methods used in literature data are not always state of the art and validated for HMO quantification. Therefore:

CONFIDENTIAL
The contents of this document constitute a trade secret and the intellectual property rights thereto belong to Nestlé S. A., Vevey, Switzerland.
This document may not be reproduced or disclosed to third parties without prior authorisation.

- ✓ HMO analyses will be conducted on a 10 mL sample of fully expressed mature milk, corresponding to a complete feed, and taken at 1st, 2nd and 4th months.

10 TRIAL DESIGN

10.1 Type of trial

This is an open, single-centre, 1 group study.

10.2 Subjects, groups and centers

50 subjects (mothers) will be recruited. The study will be performed in one center, the Faculty of Paediatrics, National University of Singapore.

10.3 Expected study duration

The study is expected to last 4 months. Subjects selected for the study will follow a 4 month follow-up.

11 STUDY POPULATION

11.1 Description

Healthy volunteers.

11.2 Subject inclusion criteria

All subjects must comply with all the following inclusion criteria:

- Gestational age between 37 and not above 42 weeks
- Baby to be enrolled between birth and V1
- 18 years old ≤ Mother ≤ 40 years old
- 18.5 ≤ pre-pregnancy BMI ≤ 29
- Mothers willing to breastfeed for the first 4 months
- Having obtained her (or her legal representative's) informed consent.

11.3 Subject exclusion criteria

Subjects representing one or more of the following criteria are excluded from participation in the study:

- Gestational diabetes
- HTA > 140/90
- Mothers who are smokers while breast-feeding
- Subject who cannot be expected to comply with the study procedures.
- Currently participating or having participated in another clinical trial during the last 12 weeks prior to the beginning of this study.

11.4 Subject withdrawal criteria

- Mothers who stop breast-feeding during the study period.
- Mothers who are hospitalized and receive drugs that may interfere with breast-feeding.

12 STUDY PRODUCT

12.1 Study product description

Not Applicable (no product tested).

CONFIDENTIAL
The contents of this document constitute a trade secret and the intellectual property rights thereto belong to Nestlé S. A., Vevey, Switzerland.
This document may not be reproduced or disclosed to third parties without prior authorisation.

12.2 Study product administration

Not Applicable (no product tested).

12.3 Study product handling

12.3.1 Storage and distribution

Not applicable (no product tested).

12.3.2 Study product accountability and reconciliation

Not applicable (no product tested).

12.4 Concomitant diet and treatment

12.4.1 Permitted concomitant diets / treatments / medications

Subjects will be instructed to maintain a stable usual diet throughout the study.

12.4.2 Unauthorized concomitant diets / treatments / medications

All drugs that interfere with breast-feeding (e.g. opioids treatment) should be excluded.

12.4.3 Concomitant diets / treatments / medications record

- Any new medications taken during the study should be recorded.
- Food consumption will be recorded three times during the study (using the 3-day Food Diary).

13 ASSESSMENT OF EFFICACY

Not Applicable (no product tested).

14 ASSESSMENT OF SAFETY

No safety concerns are expected. Subjects are healthy mothers. The study does not involve any product administration.

Adverse events occurrence and concomitant medication records.

15 CONDUCT OF THE TRIAL

15.1 Subject recruitment

Subjects will be recruited from outpatients of the Department of Neonatology at the Faculty of Paediatrics, National University of Singapore.
After the subject or the subject's legal representative has agreed to participation by signing the consent, an inclusion checklist will be completed. Subjects will be enrolled after having fulfilled all inclusion criteria, presenting none of the exclusion criteria.

15.2 Baseline

The following data will be collected at the time of enrollment:

- *For the mother*
  - Demographic data of the subject (initials of the name, age/date of birth, height (cm), weight (kg) at child's birth & before pregnancy)
  - Mode of delivery (vaginal, caesarean section)
  - Parity (1st child, 2nd, 3rd, 4th & more)

CONFIDENTIAL
The contents of this document constitute a trade secret and the intellectual property rights thereto belong to Nestlé S. A., Vevey, Switzerland.
This document may not be reproduced or disclosed to third parties without prior authorisation.

- Blood group (including Lewis Type)
- Blood pressure <u>at the time of enrollment</u>
- Ethnic group (Chinese, Indian, Malay, Caucasian, other)
- Food habits (Vegetarian/Vegan, or not)
- Confirmation that mothers are non-smokers while breast-feeding
- Any past medical history (i.e. to cross-check absence of gestational diabetes*)*
- Record any concomitant disease & medications taken <u>at the time of enrollment</u>
- Record whether the mother is breast-feeding exclusively (and since when? 1st week, 2nd, 3rd) or mixed-feeding (since when? 1st week, 2nd, 3rd)

➢ *For the baby*

- Date of birth/gestational age (weeks)
- Gender
- Anthropometry [weight (kg) / height (cm) / head circumference (cm)] at birth

15.3 Visits

15.3.1 Visit 1 (1 month +/- 5 days)

➢ *For the mother*

- 3-day Food Diary (see **Appendix A**) completed
- Breast milk sample + specification of the breast from which milk has been extracted
- Adverse events, serious adverse events & concomitant medications

➢ *For the baby*

- Anthropometry [weight (kg) / height (cm) / head circumference (cm)] at 1 month

15.3.2 Visit 2 (2 months +/- 5 days)

➢ *For the mother*

- 3-day Food Diary completed
- Breast milk sample + specification of the breast from which milk has been extracted
- Adverse events, serious adverse events & concomitant medications

➢ *For the baby*

- Anthropometry [weight (kg) / height (cm) / head circumference (cm)] at 2 months

15.4 Final Visit (4 months +/- 7 days)

➢ *For the mother*

- 3-day Food Diary completed
- Breast milk sample + specification of the breast from which milk has been extracted
- Adverse events, serious adverse events & concomitant medications

➢ *For the baby*

- Anthropometry [weight (kg) / height (cm) / head circumference (cm)] at 4 months

CONFIDENTIAL
The contents of this document constitute a trade secret and the intellectual property rights thereto belong to Nestlé S. A., Vevey, Switzerland.
This document may not be reproduced or disclosed to third parties without prior authorisation.

15.5 **Biological samples**

Phospholipid & ganglioside analyses: Procedure for milk collection

- Milk is obtained by an electric breast pump carrying a container of adequate size to receive the full milk expression (same brand for all mothers) from one breast while the infant nurses at the other breast (this produces a satisfactory let-down even in mothers who does not let down in response to an electric pump due to absence of suckling response).
- The milk is collected until the full breast is empty so that the collective sample is representative of lipid concentration per breast for one feed (this includes foremilk).
- The milk is homogenized by simple mixing the collecting sample directly in the collection vessel of the pump before taking the two aliquots for analysis.
    - ✓ If done at home, mixing by turning the vessel by hand should be fine.
    - ✓ If done at fully equipped unit, 30 sec vortex should be enough
- Foaming should be avoided during the mixing process – a sample of 10 mL is required for this analysis (a 2nd sample of 10 mL shall be further taken for HMO analyses).
- The breast selected for the collection should also be completely emptied by nursing the baby in the previous session (as the total lipid content may be influenced by the amount of residual milk left by the infant at the previous feed, yielding less than accurate estimate of daily lipid intake). Therefore when the baby is satisfied, mother is required to apply the pump to ensure complete emptying. Any milk acquired is frozen or disregarded and not used for analytical purposes. Mother also has to take care that baby will not feed on that breast until the sampling for the study is completed. Usually, two hours later, the above-mentioned procedure is carried out for breast milk sampling.
- This is repeated during different time points during lactation phases.
- Store at 4°C up to 24h; then -20°C up to 1 week; then -80°C up to ca. 6 months

HMO analyses: Procedure for milk collection

- Sampling of milk for HMO analysis will be done as described for the sample for lipid analysis.
- A sample of 10 mL per mother and time point will be collected separately in a tube for HMO analysis (a 2nd sample of 10 mL has been previously taken for phospholipid & ganglioside analyses).
- The sample will be kept at 4°C for a maximum of 1 day and frozen at -20°C and -80°C until analysis. In order to avoid inherent enzymatic degradation of HMO in the samples it is recommended to freeze the samples as fast as possible after collection.

Phospholipid analyses: Analytical procedure

Phospholipid families (i.e. phosphatidyl choline, phosphatidyl ethanolamine, phosphatidyl inositol, phosphatidyl glycerol, sphingomyelin, and phosphatidyl serine) are separated by high performance liquid chromatography and quantified with an in-line PL-ELS 1000 evaporative light scattering detector (Giuffrida et al., 2010). All chromatography is performed at 55°C. Solvent A is composed of ammonium formiate 3 g/L and solvent B of acetonitrile/methanol (100/3 v/v). Gradient conditions for phospholipid analysis are as follows: time= 0min 1% solvent A; time=19min 30% solvent A; time=21min 30% solvent A; time=24min 1% solvent A; flow rate is 1 mL/min.
The best signal and resolution is achieved at the following ELSD conditions: evap. = 90 °C; neb = 40 °C, flow rate of N_2 = L/min.

Phospholipids are extracted according to modified Folch (Folch, 1957) extraction. Briefly, aliquots (1g) of the sample are transferred into a test tube with screw cap and mixed with 9.5 mL of

CONFIDENTIAL
The contents of this document constitute a trade secret and the intellectual property rights thereto belong to Nestlé S. A., Vevey, Switzerland.
This document may not be reproduced or disclosed to third parties without prior authorisation.

chloroform/methanol (2/1 v/v). After precise addition of 20 µL of PG internal standard solution (5mg/mL), the sample solution is put into ultrasonic bath at 40°C for 15 min. After centrifugation (1000 RCF, relative centrifugal force, for 10 min), the sample solution is filtered through 0.2µm PTFE filters into glass tubes using a vacuum manifold and elut reservoirs. The filtrate is mixed with 2 mL of potassium chloride solution (8.8 g/L) and centrifuged (1000 RCF for 10 min). The organic phases are quantitatively transferred into Extrelut vials and solvents evaporated to dryness under a nitrogen flow at 40°C. The residual lipids are redissolved in 500 µL of chloroform/methanol (9/1 v/v), filtered through 0.22µm membrane PVDF filters into conical auto sampler vials and analysed by HPLC-ELSD.

Phospholipid families are quantified using a calibration curve prepared by dissolving, 700 mg of certified milk lecithin (GmbH, Köln, Germany) and 50mg of accurately weighedphosphatidylglycerol in chloroform/methanol (9/1 v/v) in a 100 mL volumetric flask. Further dilutions to volume with chloroform/methanol (9/1 v/v) are performed to give 6 concentration levels covering the concentration range of interest. The method is under validation and the validation file will be delivered by September 2011.

Phospholipids analysis will be done at the Neotron laboratory (Italy) with appropriate equipment and validated analytical protocols.

Ganglioside analyses: Analytical procedure

Ganglioside families (GM3 and GD3) are separated by high performance liquid chromatography and quantified with an in-line triplo-quadrupole mass spectrometer (AbSciex API4000) according to Sorensen.

All chromatography is performed at room temperature and solvent A is composed of water/ methanol:/ammonium acetate (1mM) (90:10:0.1M (w/w/v)) and solvent B of methanol:/ammonium acetate (1mM) (100:0.1 M (v/v)). Gradient conditions for gangliosides analysis are as follows.

**Total time (min)**	**A (%)**	**B (%)**
0	20	80
1	5	95
13	20	80
18	20	80
19	5	95
31	20	80
36	20	80

Flow rate is 0.2mL/min.

The best signal and resolution is achieved at the following MS conditions: negative polarity, Curtain Gas: 12 psi, Ion Source Gas 1 (GS1): 40 psi, Ion Source Gas 2 (GS 2): 30 psi, IosSpray Voltage: -4000 V, Temperature: 250°C, Declustering Potential: -40V, Entrance Potential: -10V and Collision Cell Exit Potential: -15 V.

Gangliosides are extracted according to modified Sorensen (Sorensen, 2006) extraction. Briefly, 1 mL of human milk is mixed with 4 mL of deminerilsed water at 40°C± 5°C. The sample solution is extracted with 10.5 mL of methanol and 5.5 mL of chloroform, mixed with 3.5 mL of water and centrifuged at 3000g for 5 minutes. The supernatant is transferred as quantitative as possible into 50 mL centrifuge tube. The residue is extracted with 2 mL of demineralised water, 5 mL methanol, 2.75 mL chloroform, let stand for 30 min and mixed with1.75 mL of deminerilsed water and centrifuged at 3000g for 5 minutes. The supernatants are combined, the volume is adjusted to 50 mL with methanol 60% and mixed with1 mL of 30 mM Na_2HPO_4 - pH 9.2.Lipid extract is applied to SPE C18 cartridges (already conditioned with 3 mL methanol and 1 mL Methanol 60%) and gangliosides are eluted with methanol.

CONFIDENTIAL
The contents of this document constitute a trade secret and the intellectual property rights thereto belong to Nestlé S. A., Vevey, Switzerland.
This document may not be reproduced or disclosed to third parties without prior authorisation.

Gangliosides families are quantified following the MS/MS transitions of GD3 producing m/z 290 product ions using the method of the standard addition.

The method is under validation and the validation file will be delivered by September 2011.
Gangliosides analysis will be done at the Neotron laboratory (Italy) with appropriate equipment and validated analytical protocols.

HMO analyses: Analytical procedure

For HMO analysis the milk sample will be defatted (if necessary). Thereafter, the samples will be subjected to a fluorescence labeling procedure with 2-aminobenzamide (Benet et al., 2011). The most abundant HMO will be identified and quantified by separation of the labeled HMO using UHPLC (Ultra High Pressure Liquid Chromatography) coupled with a fluorescence and mass detector. Equally, total HMO will be determined using the same method.

HMO analysis will be done at the Nestlé Research Centre with appropriate equipment and analytical protocols.

15.6 Follow-up

Adverse events are illnesses, signs or symptoms occurring or worsening in the course of the study. Adverse events can be serious or minor. They may or may not lead to the withdrawal of the subject from the study. In the case of a serious adverse event(s) persisting beyond trial termination, a follow-up visit may be required.

16 DATA MANAGEMENT

This Study will implement the use of a customized secure electronic Case Report Form (eCRF) database.
Patient identifiers (e.g. names, hospital ID numbers) will be removed prior to data input.

16.1 Data collection

All data captured by the study staff will be directly entered into a secured web-based database (electronic case report forms). Data will be entered from the source document into a web database (INFORM™ from Phase Forward). Hardcopy source documentation data will be used to confirm electronic data at monitoring visits and at completion of study by the study monitor. All other data should be checked for accuracy versus the subject/patient record.

Additionally to the data recorded in the eCRF, the following data will be manually loaded:

- 3-day Food Diary. The document will be kept as source document and the questionnaire will be later entered manually for further analysis by the involved scientists at the Nestlé Research Centre.
- Milk collection results will be loaded in a separate database (instructions will be given to the laboratory for recording of results in a convenient format for further data management).

On study completion, eCRF database will be forwarded for analysis. An electronic copy of the study data (i.e. DVD) will be provided to the site.

16.2 Computerized edit checks

The Data Manager will program computerized edit checks at least for data on primary outcome in order to detect discrepancies.

The Investigator will directly answer interactive discrepancies indicated by the computerised edit checks in the e-CRF. Also if necessary, manual queries can be sent to the Investigator for explanations.

CONFIDENTIAL
The contents of this document constitute a trade secret and the intellectual property rights thereto belong to Nestlé S. A., Vevey, Switzerland.
This document may not be reproduced or disclosed to third parties without prior authorisation.

16.3 Access rights

Each individual involved in the management of data for this study will be provided with a personal username and password which will not be shared with anyone. The system is done in accordance with e-signature laws (especially 21CFR part 11), meaning that saving the data is equivalent to a handwritten signature.

16.4 Audit trail

The Clinical Data Management System (INFORM^{TM}) complies with Good Clinical Practice (GCP) predicate rule requirements, laws and regulations (Personal data protection) for clinical trial and allows an audit of actions performed by users.

17 STATISTICS

17.1 Background

Many studies discuss human milk composition, but no study so far aimed at establishing normal ranges for individual components (e.g. phospholipids, gangliosides…).

17.2 Effects to be estimated

The aim of this observational study is not to estimate any effect, but to estimate normal ranges of phospholipids and gangliosides in the breast milk of healthy lactating mothers, at 1, 2 and 4 months after delivery.

17.3 Interim Analysis

In this purely observational study, interim analyses for the primary outcomes can be performed at any time, without compromising the final analysis.

17.4 Sample size calculations

50 subjects are sufficient to check the normality assumption and to estimate normal ranges if normality assumption holds or to estimate median and percentiles (10%-steps) in case of non-normal distributions of primary outcomes (before and after usual Box-Cox transformations).

17.5 Randomization

This is a purely observational study. Randomization is therefore not applicable.

17.6 Datasets to be analyzed

17.6.1 Full analysis dataset

The full analysis dataset is composed of all mothers who completed the first visit (1 month).

17.6.2 PP analysis dataset

The per-protocol (PP) analysis dataset is composed of all mothers who comply with all inclusion and exclusion criteria and who completed all visits.

17.7 Statistical analysis

All analyses will be performed on the full analysis dataset. PP-dataset only serves the purpose of sensitivity analysis for primary outcomes.

17.7.1 Primary analysis

The primary analysis is performed on primary outcomes (phospholipids and gangliosides) and consists in checking the normality assumption and to estimate normal ranges if normality

CONFIDENTIAL
The contents of this document constitute a trade secret and the intellectual property rights thereto belong to Nestlé S. A., Vevey, Switzerland.
This document may not be reproduced or disclosed to third parties without prior authorisation.

assumption holds or to estimate median and percentiles (10%-steps) in case of non-normal distributions (before and after usual Box-Cox transformations). This analysis is done separately at 1, 2 and 4 months.

17.7.2 Key secondary analyses

Key secondary analyses consist in:

- Similar analysis than primary, but for secondary outcomes (mainly oligosaccharides).
- Estimating significance of trends over time (1 to 4 months) using GLM.
- Explore relations with potential confounders (e.g. mother's age, blood group, ethnicity, BMI and food habits, as well as infant's gender or BMI) using appropriate models or subgroup analyses.

18 HANDLING OF ADVERSE EVENTS

18.1 Definition: Adverse event

An adverse event is defined as any untoward occurrence in a patient or clinical investigation subject administered an investigational product and which does not necessarily have to have a causal relationship with this treatment.

Adverse events are illnesses, signs or symptoms (including an abnormal laboratory finding) occurring or worsening in the course of the study. Adverse events can be serious or non-serious. They may or may not lead to the withdrawal of the subject / patient from the study. All adverse events must be documented and assessed for relationship to the study product.

Investigators must know and record the following information about adverse events:

- Subject and date
- Description of event
- Reporting source
- Suspect product
- Duration
- Frequency
- Intensity
- Seriousness
- Action taken
- Outcome and sequel
- Relationship to test product

18.2 Intensity

Mild: Symptoms hardly perceived, only slight impairment of general well-being.

Moderate: Clearly noticeable symptom, but tolerable without immediate relief.

Severe: Overwhelming discomfort.

18.3 Seriousness

A **serious adverse** event is any untoward medical occurrence that at any dose:

- results in death,
- is life-threatening,
- requires inpatient hospitalization or prolongation of existing hospitalization,
- results in persistent or significant disability / incapacity,
- is a congenital anomaly / birth defect, or

CONFIDENTIAL
The contents of this document constitute a trade secret and the intellectual property rights thereto belong to Nestlé S. A., Vevey, Switzerland.
This document may not be reproduced or disclosed to third parties without prior authorisation.

- is otherwise medically significant.

Non-serious: all other adverse events not corresponding to the definition of serious adverse event, are considered as non-serious.

18.4 Unexpected or expected SAE

Adverse event must be assessed as to whether they were expected to occur or not. The evaluation of expectedness is based on current knowledge and applicable product information and will be assessed by the Medical Safety Officer of the Sponsor. An unexpected AE is an AE in which the nature, severity, or frequency is not consistent with information about the condition under study and/or not consistent with information on the investigational product.

All adverse events that are suspected to be related to an investigational product and that are both unexpected and serious are considered to be SUSARs (Suspected Unexpected Serious Adverse Reactions). A SUSAR is to be reported to the regulatory authority within short notice.

18.5 Relation with test product

The reporting health care professional will assess the possibility of a link between the study product and an adverse event on the basis of the following criteria:

Unrelated: There is an **evident** other explanation for the AE, e.g.:

- the AE is obviously explained by the patient's disease;
- the AE is in accordance with the effect or adverse effect of the concomitant medication;
- the AE has occurred already prior to the administration of the study product.

Unlikely relation: Reasonable temporal relationship with the intake of the study products, **but**

- there is another plausible explanation for the occurrence of the AE.

Probable relation: Reasonable temporal relationship with the intake of the study product **and**

- plausible reasons point to a causal relationship with the study product.

Certain relation: Reasonable temporal relationship with the intake of the study product **and**

- there is no other explanation for the AE, **and**
- subsidence or disappearance of the AE on withdrawal of the study product (dechallenge), **and**
- recurrence of the symptoms on rechallenge.

18.6 Reporting and Documentation

Serious adverse event

The Clinical Safety Manager must be notified of all serious adverse events within 24 hours per fax or scan. Notification does not depend on whether there is a causal relationship with the study product or not.

SAE will be automatically emailed to: sae.declaration@rdls.nestle.com (Clinical Safety Manager), julie.chambard@rdsg.nestle.com (CPM), emilie.ba@rdls.nestle.com (DM).

A first e-mail will be sent when the description and onset date is recorded. A second e-mail will be sent when the end date is recorded. The investigator must electronically sign each SAE.

Non-serious adverse event

All adverse events must be documented on the appropriate pages of the case report forms (AE).

CONFIDENTIAL
The contents of this document constitute a trade secret and the intellectual property rights thereto belong to Nestlé S. A., Vevey, Switzerland.
This document may not be reproduced or disclosed to third parties without prior authorisation.

18.7 **Follow up**

All SAEs must be followed up until the SAE outcome is known.

In the case of a serious adverse event(s) persisting beyond trial termination, a follow up visit may be required. Further, in the event that further analyses are required for the evaluation of a potential cause-effect relationship between the study product and the adverse event, all examinations and laboratory analysis and their results will be documented in the case report forms or in an attached file. Any SAE occurring within 30 days after the last study product intake will be similarly reported within 24 hours.

18.8 **Notification**

The sponsor is responsible for the ongoing safety evaluation of the investigational product(s).

The sponsor should promptly notify all concerned investigator(s)/institution(s) and the regulatory authority(ies) of findings that could affect adversely the safety of subjects, impact the conduct of the trial, or alter the IRB/IEC's approval/favorable opinion to continue the trial.

18.9 **Reporting**

The sponsor should expedite the reporting to all concerned investigator(s)/institution(s), to the IRB(S)/IEC(s), where required, and to the regulatory authority(ies) of all adverse reactions (ARs) that are both serious and unexpected.

Such expedited reports should comply with the applicable regulatory requirement(s) and with the ICH Guideline for Clinical Safety Data Management: Definitions and Standards for Expedited Reporting.

The sponsor should submit to the regulatory authority(ies) all safety updates and periodic reports, as required by applicable regulatory requirement(s).

19 LEGAL AND ETHICAL PREREQUISITES

19.1 **Legal requirements**

The study will be conducted according to the relevant legal requirements.

19.2 **Ethical aspects**

19.2.1 Protection of the subject's confidentiality

Confidentiality of all study participants will be maintained; codes for subject identification will be utilized.

19.2.2 Informed consent

Informed consent will be obtained from the potential subject prior to any study related activities and in accordance with all applicable regulatory requirements.

The Investigator and/or his/her designee will inform the subject in addition to the written informed consent about all aspects of the subject’s study participation. The written Informed Consent must be approved by the competent Ethic Committee and competent regulatory authority if applicable. Any amendments to these documents must be approved by the competent Ethic Committee and competent regulatory authority if applicable.

The Investigator and/or his/her designee and the subject and/or the subject’s legal authorized representative (guardian, next of kin, other authorized individual) must sign and date the Written Informed Consent prior to any study related activities are performed. The subject or the authorized representative must complete the printed name and enter the date of signature themselves. If an

CONFIDENTIAL
The contents of this document constitute a trade secret and the intellectual property rights thereto belong to Nestlé S. A., Vevey, Switzerland.
This document may not be reproduced or disclosed to third parties without prior authorisation.

authorized representative signs the ICF, all efforts should be made to obtain an additional signature from the subject himself/herself.

The ICF will be signed in double and the subject and/or the authorized representative obtain one original of the signed Written Informed Consent. The second original is filed with the study documents at the investigational site.

The decision to participate in the study is entirely voluntary by the subject and/or by the authorized representative. The Investigator and/or his/her designee must emphasize to the subject and/or the authorized representative that the consent to participate can be withdrawn at any time without penalty or loss of benefits to which the subject is otherwise entitled.

19.2.3 Ethics committee approval

The study protocol will be submitted by the investigator for examination to the Institutional Review Board (IRB) / Independent Ethics committee (IEC). Commencement of the clinical trial is not permitted without written approval of the ethics committee.

The IRB / IEC must be notified of all subsequent additions or changes in the study protocol. Notification of the IRB / IEC is also required in the event of a SAE during the clinical trial.

19.2.4 Declaration of Helsinki

This trial will be conducted according to the principles and rules laid down in the Declaration of Helsinki (Appendix) and its subsequent amendments.

20 QUALITY CONTROL AND QUALITY ASSURANCE

20.1 Monitoring

Regular monitoring visits by representatives of the sponsor will be made during the study.

Monitoring will begin with an initiation visit prior to study commencement to clarify all aspects of the protocol and documentation. The purpose of later visits during the implementation period will be to evaluate study progress and adherence to protocol. The monitor will check CRFs for completeness, clarity and consistency with the information in subjects file (source data checking). At the end of the trial the monitor will make a study closing visit to all sites to ensure that all documentation is complete. In all cases, it is the responsibility of the CPM / monitor to maintain subject confidentiality.

20.2 Source documents

Medical records and data listing will serve as source documents.

Data to be recorded directly on CRFs and to be considered source data will be discussed during initiation visit and defined in the monitoring plan.

20.3 Quality Control

20.3.1 Quality control of essential documents

Quality control of essential documents will be ensured by the Clinical Project Manager.

20.3.2 Co-monitoring

Co-monitoring visits will be performed by the Clinical Project Manager with the monitor as deemed necessary.

CONFIDENTIAL
The contents of this document constitute a trade secret and the intellectual property rights thereto belong to Nestlé S. A., Vevey, Switzerland.
This document may not be reproduced or disclosed to third parties without prior authorisation.

20.4 Audits and inspections

In addition to the routine monitoring procedures, a Quality Unit exits within Nestlé Research Centre. From time to time, a representative of this unit will conduct audits of clinical research activities in accordance with NRC MPs/SOPs, to evaluate compliance with GCP guidelines and regulations.

The investigator(s) is required to permit direct access to the facilities where the study took place, source documents, CRF and applicable supporting records of subject participation for audits and inspection by IRB/IEC, regulatory authorities and company authorized representatives. The investigator(s) should make every effort to be available for the audit and/ or inspections. It the investigator(s) is contacted by any regulatory authority regarding an inspection, he/she should contact Nestlé immediately.

20.5 Responsibilities of investigator

The investigators are responsible for the following:

- Obtaining the written and dated approval of the local ethics committee (and other local regulatory agency, if any) prior to the beginning of the study.
- Selection of participants in accordance with the inclusion and exclusion criteria; obtaining the written informed consent of the subject or legal guardian.
- Maintain confidentiality of subjects and potential subjects in accordance with the Declaration of Helsinki.
- Adherence to the study protocol and the spirit of Good Clinical Practice. If modification becomes necessary, the rationale will be provided in a protocol amendment signed by the investigator and sponsor for submission to the ethics committee.
- Accurate, complete and timeliness data reported to the sponsor (CRF).
- During the course of the trial, provide subjects with any newly available information which may be relevant to them.
- Identification of adverse events with notification to sponsor, ethics committee and health authorities, as applicable.
- Co-operation with monitoring visits, audits and regulatory inspections. Providing direct access to source data and documents.
- Investigator may select a second contact at their study center to assist in implementation of the study. However, in all cases the main responsibility with respect to all aspects of this implementation rest with the principal and co-investigators.
- Archiving of the Investigator's file (including the original signed informed consent forms of all subjects) for at least 15 years after the end or the termination of the trial.

21 STUDY END PROCEDURES

21.1 Premature termination of study

Should it prove necessary to discontinue the study permanently prior to completion the sponsors will notify the investigators and additional contacts, including the IRB / IEC, of the rationale. All study relevant documents will then be returned to the sponsor, and the study product will be destroyed or returned.

21.2 Termination of study

After the completion or termination of the study, the Investigator will inform the Ethical Committee of the end of the study. A certificate of study closure will be established. Every serious or unexpected adverse event, that might affect the subject’s safety, must be brought to the Ethics Committee attention, if required by his/her Ethics Committee regulations. In addition, every report concerning an adverse event that the Sponsor sends to the Investigator must be sent by the latter to his/her ERC.

CONFIDENTIAL
The contents of this document constitute a trade secret and the intellectual property rights thereto belong to Nestlé S. A., Vevey, Switzerland.
This document may not be reproduced or disclosed to third parties without prior authorisation.

All remaining human milk samples will be stored, and will not be destroyed until all analyses (including confirmatory analyses if needed) are completed, and the related scientific work is published. At that stage (and if any samples are remaining at all) the related certificates of destruction will be issued and filed in the trial master file upon destruction. Details on the analyses and destruction procedure for the leftover milk samples will be communicated to the subjects in the letter of consent, and the relevant information material will be provided to the subject during their enrolment.

CONFIDENTIAL
The contents of this document constitute a trade secret and the intellectual property rights thereto belong to Nestlé S. A., Vevey, Switzerland.
This document may not be reproduced or disclosed to third parties without prior authorisation.

22 REFERENCES

- Asakuma S, Akahori M, Kimura K, Watanabe Y, Nakamura T, Tsunemi M, Arai I, Sanai Y, Urashima T. Sialyl oligosaccharides of human colostrum: changes in concentration during the first three days of lactation. Biosci Biotechnol Biochem. 2007 Jun;71(6):1447-51.

- Asakuma S, Urashima T, Akahori M, Obayashi H, Nakamura T, Kimura K, Watanabe Y, Arai I, Sanai Y. Variation of major neutral oligosaccharides levels in human colostrum. Eur J Clin Nutr. 2008 Apr;62(4):488-94.

- Bénet T, Austin S. On-line cleanup for 2-aminobenzamide-labeled oligosaccharides. Ana Biochem 2011 doi:10.1016/j.ab.2011.03.002

- Bitman J, Wood DL, Mehta NR, Hamosh P, Hamosh M. Comparison of the phospholipid composition of breast milk from mothers of term and preterm infants during lactation. Am J Clin Nutr 1984; 40:1103-1119.

- Bitman J, Freed LM, Neville MC, Wood DL, Hamosh P, Hamosh M. Lipid composition of prepartum human mammary secretion and postpartum milk. J Pediatr Gastroenterol Nutr 1986; 5:608-615.

- Chaturvedi, P.; Warren, C. D.; Altaye, M.; Morrow, A. L.; Ruiz-Palacios, G.; Pickering, L. K.; Newburg, D. S. Fucosylated human milk oligosaccharides vary between individuals and over the course of lactation. Glycobiology 2001, 11 (5), 365-372.

- Collins SE, Jackson MB, Lammi-Keefe CJ, Jensen RG. The simultaneous separation and quantitation of human milk lipids. Lipids 1989; 24:746-749.

- Dewettinck K, Rombaut R, Thienpont N, Trung Le T, Messens K, Van Camp J. Nutritional and technological aspects of milk fat globule membrane material. International Dairy Journal 18 (2008) 436–457.

- Erney, R. M.; Malone, W. T.; Skelding, M. B.; Marcon, A. A.; Kleman-Leyer, K. M.; O'Ryan, M. L.; Ruiz-Palacios, G.; Hilty, M. D.; Pickering, L. K.; Prieto, P. A. Variability of human milk neutral oligosaccharides in a diverse population. J. Pediatr. Gastroenterol. Nutr. 2000, 30 (2), 181-192.

- ESPGHAN Committee on Nutrition. Breast-feeding: A Commentary by the ESPGHAN Committee on Nutrition. Journal of Pediatric Gastroenterology and Nutrition 49:112–125, 2009.

- Folch, J. L., & Sloane-Stanlex, G.H. J. (1957) *Biol. Chem.* **226**, 497-509.

- German JB, Freeman SL, Lebrilla CB, Mills DA. Human Milk Oligosaccharides: Evolution, Structures and Bioselectivity as Substrates for Intestinal Bacteria. Nestle Nutr Workshop Ser Pediatr Program. 2008; 62: 205–222.

- Giuffrida F., Braun M., Flück B., Cotting C., Monard F (2010) J. of AOAC INTERNATIONAL **93** (3), 948 – 955.

- Huisman M, van Beusekom CM, Lanting CI, Nijeboer HJ, Muskiet FA, Boersma ER. Triglycerides, fatty acids, sterols, mono- and disaccharides and sugar

The contents of this document constitute a trade secret and the intellectual property rights thereto belong to Nestlé S. A., Vevey, Switzerland.
This document may not be reproduced or disclosed to third parties without prior authorisation.

alcohols in human milk and current types of infant formula milk. Eur J Clin Nutr 1996; 50:255-260.

- Huston GE, Patton S. Membrane distribution in human milks throughout lactation as revealed by phospholipid and cholesterol analyses. J Pediatr Gastroenterol Nutr 1986; 5:602-607.

- Kunz, C.; Rudloff, S.; Baier, W.; Klein, N.; Strobel, S. Oligosaccharides in human milk: structural, functional, and metabolic aspects. Annu. Rev. Nutr. 2000, 20, 699-722.

- Lammi-Keefe CJ, Ferris AM, Jensen RG. Changes in human milk at 0600, 1000, 1400, 1800, and 2200 h. J Pediatr Gastroenterol Nutr 1990; 11:83-88.

- Leo F, Asakuma S, Nakamura T, Fukuda K, Senda A, Urashima T. Improved determination of milk oligosaccharides using a single derivatization with anthranilic acid and separation by reversed-phase high-performance liquid chromatography. J Chromatogr A. 2009 Feb 27; 1216(9):1520-3.

- Martin-Sosa S, Martin MJ, Castro MD, Cabezas JA, Hueso P. Lactational changes in the fatty acid composition of human milk gangliosides. Lipids 2004; 39:111-116.

- Pan XL, Izumi T. Chronological changes in the ganglioside composition of human milk during lactation. Early Hum Dev 1999; 55:1-8.

- Peterson JA, Hamosh M, Scallan CD, Ceriani RL, Henderson TR, Mehta NR, Armand M, Hamosh P. Milk fat globule glycoproteins in human milk and in gastric aspirates of mother's milk-fed preterm infants. Pediatr Res 1998; 44:499-506.

- Rueda R, Maldonado J, Gil A. Comparison of content and distribution of human milk gangliosides from Spanish and Panamanian mothers. Ann Nutr Metab 1996; 40:194-201.

- Rueda R. The role of dietary gangliosides on immunity and the prevention of infection. British Journal of Nutrition (2007), 98, Suppl. 1, S68–S73.

- Sjögren YM, Duchén K, Lindh F, Björkstén B, Sverremark-Ekström E. Neutral oligosaccharides in colostrum in relation to maternal allergy and allergy development in children up to 18 months of age. Pediatr Allergy Immunol. 2007 Feb; 18(1):20-6.

- Lambert K. Sorensen: Rapid *commun. Mass Spectrom.* 2006; 20: 3625-3633.

CONFIDENTIAL
The contents of this document constitute a trade secret and the intellectual property rights thereto belong to Nestlé S. A., Vevey, Switzerland.
This document may not be reproduced or disclosed to third parties without prior authorisation.

23 APPENDICES

APPENDIX A
THREE-DAY FOOD DIARY

Subject Name/N° - ________________________________

Three-Day Food Diary Form

Instructions:

Please fill in the diary in English. If only another language can be used, please indicate (*in English*) which one: ______________________

In order to evaluate your dietary routine and nutrient intake, we would like for you to fill out this food diary in which you will record all that you eat for three consecutive days, including one weekend day (if possible, to ensure an accurate analysis of your nutritional status). Do not try to change your diet, eat as you have been in past. All of your recordings must include the date, time of day of consumption, all foods (with ingredients listed, if possible), beverages and supplements consumed, and amounts of all foods, beverages and supplements consumed.

Please indicate the amount of all foods and beverages you consume, as well as the preparation method (i.e., grilled, fried, baked, etc.). Make sure to also record any dips, condiments or salad dressings you consume as well. Please include brand names (name of restaurant and/or brands of ingredients) of all foods, beverages and supplements, if possible.

Example of meal recording:

- *1 cup of 2% milk*
- *1 cup of spring water*
- *½ cup of grilled, wild salmon*
- *Three cups of Asian Chicken Salad*
  - *3 oz of stir fried chicken*
  - *Two handfuls of romaine lettuce*
  - *¼ cup of cabbage*
  - *Four mandarin orange slices*
  - *One small handful of crispy wonton strips*
  - *Two tablespoons of Newman's Own low fat sesame ginger dressing*
  - *One small handful of candied walnuts*

CONFIDENTIAL
The contents of this document constitute a trade secret and the intellectual property rights thereto belong to Nestlé S. A., Vevey, Switzerland.
This document may not be reproduced or disclosed to third parties without prior authorisation.

FOOD DIARY: DAY ONE – Date: / /

**Breakfast**<br><br>Time Eaten:______	
**Snack(s)**<br><br>Time Eaten:______	
**Lunch**<br><br>Time Eaten:______	
**Snack(s)**<br><br>Time Eaten:______	
**Dinner**<br><br>Time Eaten:______	
**Snack(s)**<br><br>Time Eaten:______	

CONFIDENTIAL
The contents of this document constitute a trade secret and the intellectual property rights thereto belong to Nestlé S. A., Vevey, Switzerland.
This document may not be reproduced or disclosed to third parties without prior authorisation.

FOOD DIARY: DAY TWO – Date: / /

**Breakfast**<br><br>Time Eaten:_______	
**Snack(s)**<br><br>Time Eaten:_______	
**Lunch**<br><br>Time Eaten:_______	
**Snack(s)**<br><br>Time Eaten:_______	
**Dinner**<br><br>Time Eaten:_______	
**Snack(s)**<br><br>Time Eaten:_______	

CONFIDENTIAL
The contents of this document constitute a trade secret and the intellectual property rights thereto belong to Nestlé S. A., Vevey, Switzerland.
This document may not be reproduced or disclosed to third parties without prior authorisation.

FOOD DIARY: DAY <u>THREE</u> – Date: / /

**Breakfast**<br><br>Time Eaten:______	
**Snack(s)**<br><br>Time Eaten:______	
**Lunch**<br><br>Time Eaten:______	
**Snack(s)**<br><br>Time Eaten:______	
**Dinner**<br><br>Time Eaten:______	
**Snack(s)**<br><br>Time Eaten:______	

CONFIDENTIAL
The contents of this document constitute a trade secret and the intellectual property rights thereto belong to Nestlé S. A., Vevey, Switzerland.
This document may not be reproduced or disclosed to third parties without prior authorisation.

APPENDIX B
DECLARATION OF HELSINKI

WORLD MEDICAL ASSOCIATION DECLARATION OF HELSINKI
Ethical Principles for Medical Research Involving Human Subjects

Adopted by the 18th WMA General Assembly, Helsinki, Finland, June 1964, and amended by the:
29th WMA General Assembly, Tokyo, Japan, October 1975
35th WMA General Assembly, Venice, Italy, October 1983
41st WMA General Assembly, Hong Kong, September 1989
48th WMA General Assembly, Somerset West, Republic of South Africa, October 1996
52nd WMA General Assembly, Edinburgh, Scotland, October 2000
53th WMA General Assembly, Washington 2002 (Note of Clarification on paragraph 29 added)
55th WMA General Assembly, Tokyo 2004 (Note of Clarification on Paragraph 30 added)
59th WMA General Assembly, Seoul, October 2008

A. INTRODUCTION

1. The World Medical Association (WMA) has developed the Declaration of Helsinki as a statement of ethical principles for medical research involving human subjects, including research on identifiable human material and data.

   The Declaration is intended to be read as a whole and each of its constituent paragraphs should not be applied without consideration of all other relevant paragraphs.

2. Although the Declaration is addressed primarily to physicians, the WMA encourages other participants in medical research involving human subjects to adopt these principles.

3. It is the duty of the physician to promote and safeguard the health of patients, including those who are involved in medical research. The physician's knowledge and conscience are dedicated to the fulfillment of this duty.

4. The Declaration of Geneva of the WMA binds the physician with the words, "The health of my patient will be my first consideration," and the International Code of Medical Ethics declares that, "A physician shall act in the patient's best interest when providing medical care."

5. Medical progress is based on research that ultimately must include studies involving human subjects. Populations that are underrepresented in medical research should be provided appropriate access to participation in research.

6. In medical research involving human subjects, the well-being of the individual research subject must take precedence over all other interests.

7. The primary purpose of medical research involving human subjects is to understand the causes, development and effects of diseases and improve preventive, diagnostic and therapeutic interventions (methods, procedures and treatments). Even the best current interventions must be evaluated continually through research for their safety, effectiveness, efficiency, accessibility and quality.

8. In medical practice and in medical research, most interventions involve risks and burdens.

9. Medical research is subject to ethical standards that promote respect for all human subjects and protect their health and rights. Some research populations are particularly vulnerable and need special protection. These include those who cannot give or refuse consent for themselves and those who may be vulnerable to coercion or undue influence.

10. Physicians should consider the ethical, legal and regulatory norms and standards for research involving human subjects in their own countries as well as applicable international norms and standards. No national or international ethical, legal or regulatory requirement should reduce or eliminate any of the protections for research subjects set forth in this Declaration.

B. BASIC PRINCIPLES FOR ALL MEDICAL RESEARCH

CONFIDENTIAL
The contents of this document constitute a trade secret and the intellectual property rights thereto belong to Nestlé S. A., Vevey, Switzerland.
This document may not be reproduced or disclosed to third parties without prior authorisation.

11. It is the duty of physicians who participate in medical research to protect the life, health, dignity, integrity, right to self-determination, privacy, and confidentiality of personal information of research subjects.
12. Medical research involving human subjects must conform to generally accepted scientific principles, be based on a thorough knowledge of the scientific literature, other relevant sources of information, and adequate laboratory and, as appropriate, animal experimentation. The welfare of animals used for research must be respected.
13. Appropriate caution must be exercised in the conduct of medical research that may harm the environment.
14. The design and performance of each research study involving human subjects must be clearly described in a research protocol. The protocol should contain a statement of the ethical considerations involved and should indicate how the principles in this Declaration have been addressed. The protocol should include information regarding funding, sponsors, institutional affiliations, other potential conflicts of interest, incentives for subjects and provisions for treating and/or compensating subjects who are harmed as a consequence of participation in the research study. The protocol should describe arrangements for post-study access by study subjects to interventions identified as beneficial in the study or access to other appropriate care or benefits.
15. The research protocol must be submitted for consideration, comment, guidance and approval to a research ethics committee before the study begins. This committee must be independent of the researcher, the sponsor and any other undue influence. It must take into consideration the laws and regulations of the country or countries in which the research is to be performed as well as applicable international norms and standards but these must not be allowed to reduce or eliminate any of the protections for research subjects set forth in this Declaration. The committee must have the right to monitor ongoing studies. The researcher must provide monitoring information to the committee, especially information about any serious adverse events. No change to the protocol may be made without consideration and approval by the committee.
16. Medical research involving human subjects must be conducted only by individuals with the appropriate scientific training and qualifications. Research on patients or healthy volunteers requires the supervision of a competent and appropriately qualified physician or other health care professional. The responsibility for the protection of research subjects must always rest with the physician or other health care professional and never the research subjects, even though they have given consent.
17. Medical research involving a disadvantaged or vulnerable population or community is only justified if the research is responsive to the health needs and priorities of this population or community and if there is a reasonable likelihood that this population or community stands to benefit from the results of the research.
18. Every medical research study involving human subjects must be preceded by careful assessment of predictable risks and burdens to the individuals and communities involved in the research in comparison with foreseeable benefits to them and to other individuals or communities affected by the condition under investigation.
19. Every clinical trial must be registered in a publicly accessible database before recruitment of the first subject.
20. Physicians may not participate in a research study involving human subjects unless they are confident that the risks involved have been adequately assessed and can be satisfactorily managed. Physicians must immediately stop a study when the risks are found to outweigh the potential benefits or when there is conclusive proof of positive and beneficial results.
21. Medical research involving human subjects may only be conducted if the importance of the objective outweighs the inherent risks and burdens to the research subjects.
22. Participation by competent individuals as subjects in medical research must be voluntary. Although it may be appropriate to consult family members or community leaders, no competent individual may be enrolled in a research study unless he or she freely agrees.
23. Every precaution must be taken to protect the privacy of research subjects and the confidentiality of their personal information and to minimize the impact of the study on their physical, mental and social integrity.
24. In medical research involving competent human subjects, each potential subject must be adequately informed of the aims, methods, sources of funding, any possible conflicts of interest, institutional affiliations of the researcher, the anticipated benefits and potential risks of the study and the discomfort it may entail, and any other relevant aspects of the study. The potential subject must be informed of the right to refuse to participate in the study or to withdraw consent to participate at any time without reprisal. Special attention should be given to the specific information needs of individual potential subjects as well as to the methods used to deliver the information. After ensuring that the

CONFIDENTIAL
The contents of this document constitute a trade secret and the intellectual property rights thereto belong to Nestlé S. A., Vevey, Switzerland.
This document may not be reproduced or disclosed to third parties without prior authorisation.

potential subject has understood the information, the physician or another appropriately qualified individual must then seek the potential subject's freely-given informed consent, preferably in writing. If the consent cannot be expressed in writing, the non-written consent must be formally documented and witnessed.

25. For medical research using identifiable human material or data, physicians must normally seek consent for the collection, analysis, storage and/or reuse. There may be situations where consent would be impossible or impractical to obtain for such research or would pose a threat to the validity of the research. In such situations the research may be done only after consideration and approval of a research ethics committee.
26. When seeking informed consent for participation in a research study the physician should be particularly cautious if the potential subject is in a dependent relationship with the physician or may consent under duress. In such situations the informed consent should be sought by an appropriately qualified individual who is completely independent of this relationship.
27. For a potential research subject who is incompetent, the physician must seek informed consent from the legally authorized representative. These individuals must not be included in a research study that has no likelihood of benefit for them unless it is intended to promote the health of the population represented by the potential subject, the research cannot instead be performed with competent persons, and the research entails only minimal risk and minimal burden.
28. When a potential research subject who is deemed incompetent is able to give assent to decisions about participation in research, the physician must seek that assent in addition to the consent of the legally authorized representative. The potential subject's dissent should be respected.
29. Research involving subjects who are physically or mentally incapable of giving consent, for example, unconscious patients, may be done only if the physical or mental condition that prevents giving informed consent is a necessary characteristic of the research population. In such circumstances the physician should seek informed consent from the legally authorized representative. If no such representative is available and if the research cannot be delayed, the study may proceed without informed consent provided that the specific reasons for involving subjects with a condition that renders them unable to give informed consent have been stated in the research protocol and the study has been approved by a research ethics committee. Consent to remain in the research should be obtained as soon as possible from the subject or a legally authorized representative.
30. Authors, editors and publishers all have ethical obligations with regard to the publication of the results of research. Authors have a duty to make publicly available the results of their research on human subjects and are accountable for the completeness and accuracy of their reports. They should adhere to accepted guidelines for ethical reporting. Negative and inconclusive as well as positive results should be published or otherwise made publicly available. Sources of funding, institutional affiliations and conflicts of interest should be declared in the publication. Reports of research not in accordance with the principles of this Declaration should not be accepted for publication.

C. ADDITIONAL PRINCIPLES FOR MEDICAL RESEARCH COMBINED WITH MEDICAL CARE

31. The physician may combine medical research with medical care only to the extent that the research is justified by its potential preventive, diagnostic or therapeutic value and if the physician has good reason to believe that participation in the research study will not adversely affect the health of the patients who serve as research subjects.
32. The benefits, risks, burdens and effectiveness of a new intervention must be tested against those of the best current proven intervention, except in the following circumstances:
    - The use of placebo, or no treatment, is acceptable in studies where no current proven intervention exists; or
    - Where for compelling and scientifically sound methodological reasons the use of placebo is necessary to determine the efficacy or safety of an intervention and the patients who receive placebo or no treatment will not be subject to any risk of serious or irreversible harm. Extreme care must be taken to avoid abuse of this option.
33. At the conclusion of the study, patients entered into the study are entitled to be informed about the outcome of the study and to share any benefits that result from it, for example, access to interventions identified as beneficial in the study or to other appropriate care or benefits.
34. The physician must fully inform the patient which aspects of the care are related to the research. The refusal of a patient to participate in a study or the patient's decision to withdraw from the study must never interfere with the patient-physician relationship.

CONFIDENTIAL
The contents of this document constitute a trade secret and the intellectual property rights thereto belong to Nestlé S. A., Vevey, Switzerland.
This document may not be reproduced or disclosed to third parties without prior authorisation.

35. In the treatment of a patient, where proven interventions do not exist or have been ineffective, the physician, after seeking expert advice, with informed consent from the patient or a legally authorized representative, may use an unproven intervention if in the physician's judgment it offers hope of saving life, re-establishing health or alleviating suffering. Where possible, this intervention should be made the object of research, designed to evaluate its safety and efficacy. In all cases, new information should be recorded and, where appropriate, made publicly available.

22.10.2008

CONFIDENTIAL
The contents of this document constitute a trade secret and the intellectual property rights thereto belong to Nestlé S. A., Vevey, Switzerland.
This document may not be reproduced or disclosed to third parties without prior authorisation.
