# Supplementary material for: Longitudinal change of selected human milk oligosaccharides and association to infants’ growth, an observatory, single center, longitudinal cohort study
Source: PLoS One. 2017 Feb 9;12(2):e0171814. doi: 10.1371/journal.pone.0171814 (PMC5300226; doi:10.1371/journal.pone.0171814)
Supplement: S1 Table — (PDF) [file pone.0171814.s005.pdf]

raw data

ID	sex	mode of delivery	body length	body weight	head circumference
			birth	birth	birth
001	Female	caesarean section	50	3.165	33.5
002	Male	vaginal	50.5	3.735	34
003	Male	vaginal	51	3.4	33.5
004	Male	vaginal	51	3.125	33.5
005	Male	vaginal	50.5	3.36	34
006	Female	vaginal	49	2.95	33.5
007	Male	caesarean section	53	3.275	34
008	Female	caesarean section	49	3.46	36
009	Female	caesarean section	51	3.03	34
010	Female	caesarean section	50	3.66	35.5
011	Female	caesarean section	51	3.19	35
012	Female	caesarean section	53	3.29	35
013	Male	vaginal	52	3.49	33.5
014	Female	caesarean section	51	3.365	33
015	Female	vaginal	50.5	3.295	34
016	Male	vaginal	53	3.765	35.5
017	Female	vaginal	50	3.495	34
018	Male	vaginal	53	3.84	33
019	Female	vaginal	48	2.965	32
020	Female	caesarean section	47	2.28	31.5
021	Male	vaginal	46	2.495	32
022	Female	caesarean section	47	2.845	32
023	Male	vaginal	47	2.91	33
024	Male	vaginal	51	3.69	36
025	Male	vaginal	53	3.55	33
026	Male	vaginal	51	3.75	34
027	Male	vaginal	48	3.105	33
028	Female	vaginal	46	3.095	34.5
029	Male	vaginal	54	3.64	35.5
030	Female	vaginal	50	2.855	32.5
031	Female	caesarean section	50	3.255	35
032	Female	caesarean section	50	3.33	34
033	Male	vaginal	48	3.415	33
034	Male	caesarean section	52	3.1	32.5
035	Female	vaginal	51	3.175	34
036	Male	caesarean section	50	2.98	34
037	Female	vaginal	53	3.65	35
038	Female	vaginal	45	2.34	33
039	Male	caesarean section	50	3.185	34
040	Male	caesarean section	52	3.59	35
041	Female	vaginal	50	3.048	35
042	Female	caesarean section	51	3.485	35
043	Female	caesarean section	55	3.595	35.5
044	Female	vaginal	50	2.81	33
045	Female	vaginal	49	2.68	31
046	Male	caesarean section	54	3.65	33.5
047	Male	vaginal	53	3.89	33
048	Male	vaginal	50	3.04	33.5
049	Male	vaginal	51.5	3.925	36
050	Male	vaginal	50	3.175	34.5

body length 1 month	body weight 1 month	head circumference 1 month	body length 2 month	body weight 2 month	head circumference 2 month
55	4.482	37	58.5	5.634	38.5
59	4.86	39	61	6.35	40
56	4.574	38	58	5.63	40
54	4.5	37	59	5.455	39.5
57	4.616	38	59	5.63	39.5
54	4.036	36	58	5.134	37.5
55.5	4.534	38.5	61	6.034	40
57	4.61	38	57	5.49	40
54	3.93	37	58	5.42	40
53	3.768	37	57	5.062	38
55	3.935	37	59	5.14	39
56	3.945	37	59	4.89	39
57	4.58	38	60	5.985	39
57	4.75	37	61	5.98	38
54	4.25	37.5	58	4.52	39
59	5.51	39	63	7.192	41
58	5.015	37.5	61	5.98	38.5
59	5.135	38	62	6.01	39
52	3.46	35.5	56	4.43	37
50.5	3.16	35	56	4	36.5
53	3.65	36.5	57	4.944	38.5
53	3.872	35.5	56	5.208	37
54.5	3.988	37	58	4.86	39
56	5.118	38	57	6.076	40
57	4.65	39.5	62	6.194	41
54	4.62	36	60	6.012	40
53	4.318	38	56	5.32	39
54.5	3.766	37.5	55	4.714	39
57	5.15	37	60	5.886	38
54	3.62	35.5	57	4.944	38
55	4.08	38.5	57.5	4.98	40
55	3.56	36	57	4.356	38
53	4.82	38	58.5	5.55	40
55	4.052	37	58	4.994	39.8
53	3.58	37.5	56	5.1	39.5
55	3.882	36.5	61	5.758	39.5
57	4.69	38	60	6.25	41
51	3.3	35	53	4.06	37
54	4.202	37	58	5.938	40
56	4.436	37.5	59	5.528	38.5
55.5	3.736	37.5	59	4.824	38.5
57	4.715	40	59	5.84	41
57	4.902	37.5	60	5.99	39
52	3.778	35	55	5.075	37.5
52.5	3.782	35	55.5	4.936	36.5
56	5.17	39	61	6.685	41
57	4.865	41	62	6.372	42
54	4.032	36.5	59	5.704	38.5
55	5.475	39	60	6.312	40
54	4.21	37	57.5	5.1	39

body length 4 month	body weight 4 month	head circumference 4 month	BMI birth	BMI 1 month	BMI 2 months
63	6.896	40.5	12.7	14.8	16.5
67	8.53	44	14.6	14.0	17.1
62	6.845	42	13.1	14.6	16.7
63	6.485	42	12.0	15.4	15.7
66.5	8	43	13.2	14.2	16.2
63	6.458	40	12.3	13.8	15.3
66	7.445	43	11.7	14.7	16.2
63	6.918	43.5	14.4	14.2	16.9
65	7.09	41	11.6	13.5	16.1
62	6.53	40.5	14.6	13.4	15.6
64	6.786	42	12.3	13.0	14.8
64	6.545	41	11.7	12.6	14.0
66	8.1	43.5	12.9	14.1	16.6
67	7.73	41	12.9	14.6	16.1
63	6.048	40.5	12.9	14.6	13.4
66.5	8.22	43	13.4	15.8	18.1
67	6.748	41	14.0	14.9	16.1
66	7.256	41	13.7	14.8	15.6
60.5	5.828	38.5	12.9	12.8	14.1
59	4.935	38	10.3	12.4	12.8
61	6.052	39	11.8	13.0	15.2
60.5	6.476	39	12.9	13.8	16.6
64	6.335	42	13.2	13.4	14.4
64	7.78	42	14.2	16.3	18.7
69	8.34	45	12.6	14.3	16.1
65	7.335	42	14.4	15.8	16.7
62	6.992	41.5	13.5	15.4	17.0
60	6.082	41	14.6	12.7	15.6
67	7.378	41	12.5	15.9	16.4
62	6.432	40.5	11.4	12.4	15.2
62	6.724	42	13.0	13.5	15.1
61	5.358	40	13.3	11.8	13.4
61	7.314	41.5	14.8	17.2	16.2
63	6.366	42	11.5	13.4	14.8
62	7.322	42.5	12.2	12.7	16.3
67	7.784	42.5	11.9	12.8	15.5
67	8.324	43	13.0	14.4	17.4
58	5.516	41	11.6	12.7	14.5
65	7.822	43	12.7	14.4	17.7
62	7.012	41	13.3	14.1	15.9
64	6.442	42	12.2	12.1	13.9
64	7.368	44	13.4	14.5	16.8
65	7.634	42	11.9	15.1	16.6
59	5.902	39.5	11.2	14.0	16.8
60	5.438	39.5	11.2	13.7	16.0
65	8.42	43	12.5	16.5	18.0
67	8.02	43	13.8	15.0	16.6
63	7.05	40.5	12.2	13.8	16.4
64	7.844	43	14.8	18.1	17.5
60	6.248	41	12.7	14.4	15.4

BMI 4 months	2'FL mg/l 1 month	2'FL mg/l 2 months	2'FL mg/l 4 months	LNnT mg/l 1 month	LNnT mg/l 2 months	LNnT mg/l 4 months	LNT mg/l 1 month	LNT mg/l 2 months
17.4	32	23	ND	157	59	ND	1088	618
19.0	2864	2236	1765	362	172	82	678	372
17.8	2088	2331	2419	294	283	302	710	722
16.3	15	16	10	64	119	81	881	869
18.1	122	50	15	202	87	38	790	423
16.3	1209	843	781	417	224	182	1527	691
17.1	2513	1665	1499	130	59	22	916	471
17.4	1563	1364	829	130	100	51	904	659
16.8	27	14	10	158	70	49	1801	1220
17.0	3281	1836	1288	275	144	84	924	462
16.6	3209	1945	n/a	209	105	n/a	670	819
16.0	1718	1629	1414	203	130	60	768	517
18.6	2734	2125	1615	172	97	52	833	585
17.2	8	10	12	130	118	32	1533	1339
15.2	43	25	16	336	189	131	927	1043
18.6	44	37	22	268	143	90	1004	515
15.0	1751	2005	1277	233	194	105	390	521
16.7	2416	2105	1519	261	141	49	460	415
15.9	1390	1059	917	388	208	133	1290	619
14.2	2341	1772	1470	197	121	69	587	547
16.3	1492	1524	764	378	207	94	899	542
17.7	3310	3290	1907	219	235	110	627	599
15.5	15	21	15	214	56	81	1607	737
19.0	2106	1627	1209	331	121	30	1337	477
17.5	1189	1059	677	208	128	100	1735	1003
17.4	14	10	5	165	39	23	2491	810
18.2	3579	2836	2635	261	177	137	875	514
16.9	7	10	5	149	103	60	2041	1227
16.4	9	6	4	139	63	16	2094	1132
16.7	13	12	12	212	152	126	1166	906
17.5	1363	1039	712	169	118	45	1399	1039
14.4	1794	1640	1468	534	397	353	1596	1373
19.7	1207	950	927	285	221	87	2185	1836
16.0	1557	1204	1186	234	166	98	1242	1011
19.0	2017	1573	712	158	86	61	613	236
17.3	1853	1907	1306	229	144	110	1166	555
18.5	2682	1727	1511	489	224	204	931	312
16.4	32	24	15	310	202	123	1318	974
18.5	2132	1827	1494	124	56	20	977	565
18.2	1522	1587	1348	130	46	40	824	294
15.7	1588	1564	993	304	196	73	888	462
18.0	2217	1909	1520	274	177	103	1016	721
18.1	16	11	7	192	124	67	924	672
17.0	1322	1062	878	211	147	103	1148	385
15.1	4990	3863	3505	322	233	216	452	361
19.9	1682	1367	1260	260	273	211	1132	892
17.9	2387	1978	1590	316	184	127	988	594
17.8	2715	1532	1012	225	130	64	584	337
19.2	12	18	10	155	99	47	1178	783
17.4	16	15	7	174	127	23	2762	2254

LNT mg/l 4 months	3'SL mg/l 1 month	3'SL mg/l 2 months	3'SL mg/l 4 months	6'SL mg/l 1 month	6'SL mg/l 2 months	6'SL mg/l 4 months
3	209	363	26	542	212	ND
303	198	207	193	742	392	148
693	186	227	260	308	184	90
804	206	156	228	432	266	172
257	134	179	179	590	310	142
499	183	260	229	337	103	59
272	227	157	143	613	246	112
325	221	188	157	611	319	139
1084	176	257	215	425	259	150
275	157	124	112	889	348	132
n/a	303	328	n/a	799	352	n/a
330	297	264	320	381	180	71
535	126	92	167	491	170	116
789	242	203	191	470	273	158
985	309	477	462	505	357	340
385	336	161	215	347	152	96
326	161	298	269	325	283	97
248	83	87	116	240	137	46
449	275	201	268	653	341	123
465	351	284	219	826	500	181
274	244	205	237	464	223	92
245	207	217	325	685	579	184
819	337	222	181	581	376	189
264	437	304	295	1000	386	96
707	296	240	182	507	333	111
421	217	244	258	428	125	56
547	164	177	108	391	174	87
799	427	238	278	516	228	72
659	333	274	221	575	299	142
986	168	231	226	596	355	198
444	175	170	189	881	483	172
906	173	161	194	208	68	69
1024	141	205	219	646	284	109
372	226	186	220	417	181	58
151	243	175	130	474	212	82
362	276	188	157	634	387	137
380	155	104	98	500	166	91
716	153	130	110	384	178	88
259	250	170	175	807	395	160
253	268	215	180	599	341	217
188	116	104	149	243	175	70
265	170	214	220	421	270	100
538	241	211	224	389	195	79
271	236	168	183	572	223	123
397	290	170	207	658	217	154
511	117	148	177	493	249	160
616	178	148	182	589	356	197
273	247	242	246	681	268	190
479	272	228	229	440	170	162
1524	388	321	287	714	478	211
